# Global, regional, and national burden of neck pain, 1990–2020, and projections to 2050: a systematic analysis of the Global Burden of Disease Study 2021

**DOI:** 10.1016/S2665-9913(23)00321-1

**Published:** 2024-02-19

**Authors:** GBD 2021 Neck Pain Collaborators, Ai-Min Wu, Marita Cross, James M Elliott, Garland T Culbreth, Lydia M Haile, Jaimie D Steinmetz, Hailey Hagins, Jacek A Kopec, Peter M Brooks, Anthony D Woolf, Deborah R Kopansky-Giles, David M Walton, Julia M Treleaven, Karsten E Dreinhoefer, Neil Betteridge, Mitra Abbasifard, Zeinab Abbasi-Kangevari, Isaac Yeboah Addo, Miracle Ayomikun Adesina, Qorinah Estiningtyas Sakilah Adnani, Janardhana P Aithala, Fadwa Alhalaiqa Naji Alhalaiqa, Yousef Alimohamadi, Sohrab Amiri, Hubert Amu, Benny Antony, Jalal Arabloo, Aleksandr Y Aravkin, Mohammad Asghari-Jafarabadi, Gamechu Hunde Atomsa, Sina Azadnajafabad, Ahmed Y Azzam, Soroush Baghdadi, Saliu A Balogun, Asaminew Birhanu Balta, Maciej Banach, Morteza Banakar, Amadou Barrow, Azadeh Bashiri, Alehegn Bekele, Isabela M Bensenor, Pankaj Bhardwaj, Ajay Nagesh Bhat, Awraris Hailu Bilchut, Andrew M Briggs, Rachelle Buchbinder, Chao Cao, Akhilanand Chaurasia, Jesus Lorenzo Chirinos-Caceres, Steffan Wittrup McPhee Christensen, Kaleb Coberly, Ewerton Cousin, Omid Dadras, Xiaochen Dai, Katie de Luca, Azizallah Dehghan, Huan-Ji Dong, Michael Ekholuenetale, Muhammed Elhadi, Habitu Birhan Eshetu, Sharareh Eskandarieh, Farshid Etaee, Adeniyi Francis Fagbamigbe, Jawad Fares, Ali Fatehizadeh, Alireza Feizkhah, Manuela L Ferreira, Nuno Ferreira, Florian Fischer, Richard Charles Franklin, Balasankar Ganesan, Mathewos Alemu Gebremichael, Urge Gerema, Ali Gholami, Sherief Ghozy, Tiffany K Gill, Mahaveer Golechha, Pouya Goleij, Davide Golinelli, Simon Matthew Graham, Arvin Haj-Mirzaian, Netanja I Harlianto, Jan Hartvigsen, Mohammad Hasanian, Mohammed Bheser Hassen, Simon I Hay, Jeffrey J Hebert, Golnaz Heidari, Amir Human Hoveidaei, Alexander Kevin Hsiao, Segun Emmanuel Ibitoye, Chidozie C D Iwu, Louis Jacob, Manthan Dilipkumar Janodia, Yingzhao Jin, Jost B. Jonas, Charity Ehimwenma Joshua, Himal Kandel, Yousef Saleh Khader, Himanshu Khajuria, Ejaz Ahmad Khan, Moien AB Khan, Moawiah Mohammad Khatatbeh, Sorour Khateri, Hamid Reza Khayat Kashani, Mohammad Saeid Khonji, Jagdish Khubchandani, Yun Jin Kim, Adnan Kisa, Ali-Asghar Kolahi, Hamid Reza Koohestani, Kewal Krishan, Mohammed Kuddus, Ambily Kuttikkattu, Savita Lasrado, Yo Han Lee, Samson Mideksa Legesse, Stephen S Lim, Xuefeng Liu, Justin Lo, Narges Malih, Shisir Prasad Manandhar, Elezebeth Mathews, Mohamed Kamal Mesregah, Tomislav Mestrovic, Ted R Miller, Seyed Peyman Mirghaderi, Awoke Misganaw, Esmaeil Mohammadi, Shafiu Mohammed, Ali H Mokdad, Sara Momtazmanesh, Mohammad Ali Moni, Ebrahim Mostafavi, Christopher J L Murray, Tapas Sadasivan Nair, Seyed Aria Nejadghaderi, Ogochukwu Janet Nzoputam, In-Hwan Oh, Osaretin Christabel Okonji, Mayowa O Owolabi, Kevin Pacheco-Barrios, Mohammad Taha Pahlevan Fallahy, Seoyeon Park, Jay Patel, Shrikant Pawar, Paolo Pedersini, Mario F P Peres, Ionela-Roxana Petcu, Mohammadreza Pourahmadi, Ibrahim Qattea, Pradhum Ram, Mohammad-Mahdi Rashidi, Salman Rawaf, Nazila Rezaei, Negar Rezaei, Umar Saeed, Fatemeh Saheb Sharif-Askari, Saina Salahi, Monika Sawhney, Austin E Schumacher, Mahan Shafie, Saeed Shahabi, Ataollah Shahbandi, Ali Shamekh, Saurab Sharma, Rahman Shiri, Parnian Shobeiri, Ehsan Sinaei, Ambrish Singh, Jasvinder A. Singh, Paramdeep Singh, Anna Aleksandrovna Skryabina, Amanda E Smith, Mohammad Tabish, Ker-Kan Tan, Masresha Derese Tegegne, Samar Tharwat, Seyed Mohammad Vahabi, Sahel Valadan Tahbaz, Tommi Juhani Vasankari, Narayanaswamy Venketasubramanian, Stein Emil Vollset, Yuan-Pang Wang, Taweewat Wiangkham, Naohiro Yonemoto, Moein Zangiabadian, Iman Zare, Dawit T Zemedikun, Peng Zheng, Kanyin Liane Ong, Theo Vos, Lyn M March

## Abstract

**Background:**

Neck pain is a highly prevalent condition that leads to considerable pain, disability, and economic cost. We present the most current estimates of neck pain prevalence and years lived with disability (YLDs) from the Global Burden of Diseases, Injuries, and Risk Factors Study (GBD) by age, sex, and location, with forecasted prevalence to 2050.

**Methods:**

Systematic reviews identified population-representative surveys used to estimate the prevalence of and YLDs from neck pain in 204 countries and territories, spanning from 1990 to 2020, with additional data from opportunistic review. Medical claims data from Taiwan (province of China) were also included. Input data were pooled using DisMod-MR 2.1, a Bayesian meta-regression tool. Prevalence was forecast to 2050 using a mixed-effects model using Socio-demographic Index as a predictor and multiplying by projected population estimates. We present 95% UIs for every metric based on the 2·5th and 97·5th percentiles of 100 draws of the posterior distribution.

**Findings:**

Globally, in 2020, neck pain affected 203 million (95% uncertainty interval [UI] 163–253) people. The global age-standardised prevalence rate of neck pain was estimated to be 2450 (1960–3040) per 100 000 population and global age-standardised YLD rate was estimated to be 244 (165–346) per 100 000. The age-standardised prevalence rate remained stable between 1990 and 2020 (percentage change 0·2% [–1·3 to 1·7]). Globally, females had a higher age-standardised prevalence rate (2890 [2330–3620] per 100 000) than males (2000 [1600–2480] per 100 000), with the prevalence peaking between 45 years and 74 years in male and female sexes. By 2050, the estimated global number of neck pain cases is projected to be 269 million (219–322), with an increase of 32·5% (23·9–42·3) from 2020 to 2050. Decomposition analysis of the projections showed population growth was the primary contributing factor, followed by population ageing.

**Interpretation:**

Although age-standardised rates of neck pain have remained stable over the past three decades, by 2050 the projected case numbers are expected to rise. With the highest prevalence in older adults (higher in females than males), a larger effect expected in low-income and middle-income countries, and a rapidly ageing global population, neck pain continues to pose a challenge in terms of disability burden worldwide. For future planning, it is essential we improve our mechanistic understanding of the different causes and risk factors for neck pain and prioritise the consistent collection of global neck pain data and increase the number of countries with data on neck pain.

**Funding:**

Bill & Melinda Gates Foundation and Global Alliance for Musculoskeletal Health.

## Introduction

Neck pain, irrespective of traumatic or non-traumatic cause, is a common and often disabling condition, contributing to considerable self-reports of pain, disability, and ongoing personal and health-care burden worldwide.^1^, ^2^ The Global Burden of Diseases, Injuries, and Risk Factors Study (GBD) is a comprehensive analysis of health loss attributed to diseases, injuries, and risk factors across 204 countries and territories.^3^ Each iteration of the GBD has reported on the combined burden of musculoskeletal conditions in general, and on neck pain specifically,^4^ with the aim of assisting health-care providers and policy makers to identify and promote the implementation of effective assessment and intervention strategies. Reports of neck pain epidemiology have characterised neck pain on the basis of common clinical features (such as tenderness and limited mobility);^2^, ^3^, ^5^ however, it is crucial to recognise that neck pain is a heterogeneous condition clinically.^6^, ^7^

Despite the high prevalence of neck pain, causes and associated risk factors have not been clearly defined in population estimates. Evidence suggests causes are multifactorial^8^, ^9^, ^10^ and include, but are not limited to, muscle strain (including resulting from poor posture, sleep disorders, repetitive movements, or injury); lack of exercise; inflammatory and degenerative changes in the spinal discs, joints, ligaments, and nerves; non-catastrophic injury from a motor vehicle collision, work-related event, sports-related event, or other degenerative conditions (eg, secondary osteoarthritis of cervical facet joints or degenerative cervical myelopathy); or neurological conditions and neoplasms.^9^, ^11^, ^12^, ^13^

**Research in context**

**Evidence before this study**

The Global Burden of Diseases, Injuries, and Risk Factors Study (GBD) is a comprehensive source of global, regional, and country-level estimates of disease burden over time. Previous studies have reported neck pain to be a major cause of disability. To update these GBD data, a systematic review of population-representative studies of neck pain from 1980 to 2017 using the search terms “neck pain”, “neck ache”, “neckache”, and “cervical pain” individually and combined with each of the following terms: “prevalen*”, “inciden*”, “cross-sectional”, “cross sectional”, “epidemiol*”, “survey”, “population-based”, “population based”, “population study”, and “population sample”. A further search from 2017 to 2020 was also done. These data were incorporated into the GBD model to estimate the burden of neck pain in 2020.

**Added value of this study**

For the first time, this work provides projections of neck pain prevalence at the global and regional level to the year 2050. We estimated that neck pain affected 203 million people in 2020 with age-standardised prevalence higher in females than males. The number of people with neck pain is projected to increase by 32·5% (95% uncertainty interval 23·9–42·3) globally to 269 million by the year 2050. In most regions, population growth is the main contributor to the increase in number of neck pain cases, followed by population ageing.

**Implications of all the available evidence**

There remains a substantial burden from neck pain. These GBD estimates are based on few and disparate sources of data, highlighting the need to increase the collection of standardised data at the country level. The identification and evaluation of the risk factors for neck pain, such as trauma and work-related risks, are essential to determine effective prevention and management strategies to decrease this burden.

The aim of this systematic analysis was to provide an update on global, regional, and national estimates of prevalence and years lived with disability (YLDs) due to neck pain, with projections of prevalence for 2030–50.

## Methods

### Overview

This GBD study produced comprehensive estimates of neck pain prevalence and YLDs according to age, sex, location, and year in 204 countries and territories from 1990 to 2020. The Guidelines for Accurate and Transparent Health Estimates Reporting (GATHER)^14^ statement were followed (appendix p 2). Detailed methodology for the GBD study is reported elsewhere.^3^ This Article was produced as part of the GBD Collaborator Network and in accordance with the GBD Protocol.

### Case definition

The GBD reference case definition for neck pain was pain (with or without referral into the upper limb [or limbs]) that lasts for at least 1 day.^5^ The neck includes the area from the occiput to the first thoracic vertebra.

### Input data

An initial systematic review of neck pain incidence and prevalence in population-representative data sources was presented for GBD 2010^5^ and updated for GBD 2017.^3^, ^4^ A further search of data between 2017 and 2020 found an additional eight neck pain studies, which were included for this study (PRISMA diagram in the appendix p 4). All included studies are presented in the appendix (p 28). Survey sources were excluded if they were not representative of the population, did not include primary data, or captured a small sample size. Further data for this study were included from opportunistic searches and contributions of datasets by GBD Collaborators.^3^ Additionally, available medical claims data from Taiwan (province of China) for 2016 were included based on ninth and tenth revision International Classification of Diseases (ICD) coding (appendix p 4). The ICD-9 code is 723.1, and the ICD-10 code is M54.2. Each newly identified and obtained data source was given a unique identifier (NID) and included in the Global Health Data Exchange (GHDx).

The GBD study estimated neck pain prevalence in all countries; however, for most GBD disease models, input data were not available for every location. In these cases, prevalence estimates were obtained through the use of regional priors and country-level covariates. Available data were used from all countries in a given GBD region to produce regional estimates, and these priors were passed down to each country in the region to help inform country estimates. In regions with no data, estimates were informed by super-region priors. Countries for which data were available are in the appendix (pp 5–6).

Soft-tissue neck conditions, such as non-catastrophic trauma events, were assumed to be included in these prevalence studies of neck pain because there is no category in the separate GBD injury model process to identify these neck injuries. However, cervical vertebral fractures and their sequelae would be included in the GBD injury model and not be represented in the estimates for neck pain reported here.

### Data processing and disease modelling

Reported estimates of prevalence were split by age and sex where possible. If studies reported prevalence for broad age groups by sex and by specific age groups for male and female sexes combined, age-specific estimates were split by sex using the reported sex ratio and bounds of uncertainty. Data that exclusively reported male and female prevalence combined were split into sex-specific data points by running a regression on the ratio of female-to-male prevalence in the total dataset using a meta-regression tool, MR-BRT (Meta—Regression-Bayesian Regularised Trimmed; details described elsewhere^3^), then applying this ratio to data reporting male and female sexes combined. The female-to-male ratio was 1·31 (95% uncertainty interval [UI] 1·30–1·32). Additionally, data that reported prevalence in large age groups (>25 years) were split into 5-year age groups by applying the global age pattern of prevalence from the model of neck pain in GBD 2017. To estimate age-standardised rates, the standard population was calculated using the non-weighted mean of the GBD year's age-specific population proportional distributions for all national locations with a population greater than 5 million people in the GBD year.^15^ Uncertainty was estimated using 100 draws from our Bayesian model. We present 95% UIs for every metric based on the 2·5th and 97·5th percentiles of 100 draws of the posterior distribution.

In order to have the greatest data coverage possible, we accepted studies that used alternative case definitions than our reference and then adjusted for systematic bias using an adjustment factor specific to each alternative case definition. Bias adjustments were conducted using a network MR-BRT analysis for studies that reported an anatomical region beyond the defined area for the neck, episode duration greater than 3 months, recall periods of 1 week to 1 month, recall periods between 1 month and 1 year, activity-limiting pain, and studies surveying school children. Adjustment factors were calculated by pairing data with varying case definitions by year, age, sex, and location, and then estimating the logit difference between the prevalence of alternative and reference case definitions (appendix p 7). The adjustment factors reflect the ratio of the alternative over the reference case definition. Thus, for example, data for “anatomical region too broad” are divided by the adjustment factor of 2·63 (appendix p 7). After adjustment, data points with an age-adjusted prevalence rate of more than two median absolute deviations from the median by sex, year, and location were considered outliers and removed. Data inputs measured as period prevalence were adjusted in our crosswalking process to the level expected if it had been measured as point prevalence. Data were pooled using DisMod-MR 2.1. We assumed no incidence or prevalence before 5 years of age, and no risk factors for neck pain were included in the modelling process.

Neck pain estimates were then split by severity based on Medical Expenditure Panel Surveys (MEPS;^16^ appendix p 7). Respondents of the MEPS reported on reasons for health-care contact over a 2-year period, which were then coded to ICD-9. MEPS respondents reported on general health status through the 12-Item Short Form Survey (SF-12), a self-reported outcome measure assessing the impact of health on an individual's everyday life.^3^ From a series of purposive surveys among staff at the Institute for Health Metrics and Evaluation (University of Washington, Seattle, WA, USA) and participants at a GBD training workshop who completed SF-12 for 60 health states in GBD ranging from mild to very severe, a person's disability weight was predicted from a regression of the SF-12 scores and the value of the GBD disability weight for each health state. Thus, for each MEPS respondent, we derived a value of the disability weight indirectly reported on SF-12 and parsed out the contribution of other conditions by correcting for any comorbid conditions influencing an individual's experience of health loss. Respondents were then classified into five categories (the four neck pain health states with disability weights and an asymptomatic health state) taking the midpoint between disability weight values as the threshold between severity levels (appendix p 8).^3^ Disability weights were derived from pairwise comparisons of the relative severity of different health states conducted in nine countries' disability weight surveys (such as interviews with communities and stakeholders, as well as the individuals through SF-12) and an open-access web-based survey (appendix p 8).^1^, ^17^ Finally, YLD estimates underwent comorbidity correction to account for the co-occurrence of all other conditions (eg, the neck pain associated with other disorders such as rheumatoid arthritis, depression, or headache) quantified in GBD.^3^ No mortality is attributed to neck pain within the GBD model, and as a result estimates for disability-adjusted life-years (DALYs) and YLDs are identical.

### Estimate projections

Forecast global and regional cases of neck pain to the year 2050 were computed by forecasting prevalence and population estimates.^18^, ^19^ Age-specific, location-specific, and sex-specific GBD prevalence from 1990 to 2020 was logit transformed and used in the following regression model:

$$E[\log it(Y_{l,a,s,y})]=\beta_{1}SDI_{l,y}+\alpha_{l,a,s}$$

The term on the left side of the equation is the forecasted logit(prevalence), β_1_ is the fixed coefficient on Socio-demographic Index (SDI) over time, and α_l,a,s_ is the location-age-sex-specific random intercept. SDI is a composite indicator of development status strongly correlated with health outcomes.^15^ In short, it is a summary measure of the total fertility rate for females younger than 25 years, mean years of schooling for those aged 15 years and older, and lag-distributed income per capita, which models changes in consumption because of a change in disposable income. Forecasts of SDI for every year–location combination were generated from forecasts for the three underlying components: total fertility under age 25, educational attainment, and lag-distributed income.^19^ To obtain forecasted cases, forecasted rates were multiplied by forecasted population counts.^19^ Forecasted prevalence rates were intercept-shifted to GBD prevalence by subtracting forecasted estimation year 2020 prevalence rates from GBD estimation year 2020 prevalence rates and using this difference to shift all forecasted values through to the year 2050. Validation testing was done (appendix p 8). A Das Gupta decomposition analysis was performed to determine the relative contributions to the change in case number between 2020 and 2050 of population growth, population ageing, and changes in prevalence unrelated to demographics.^20^

### Role of the funding source

The funders of the study had no role in the study design, data collection, data analysis, data interpretation, or writing of the report.

## Results

**Figure 1:**
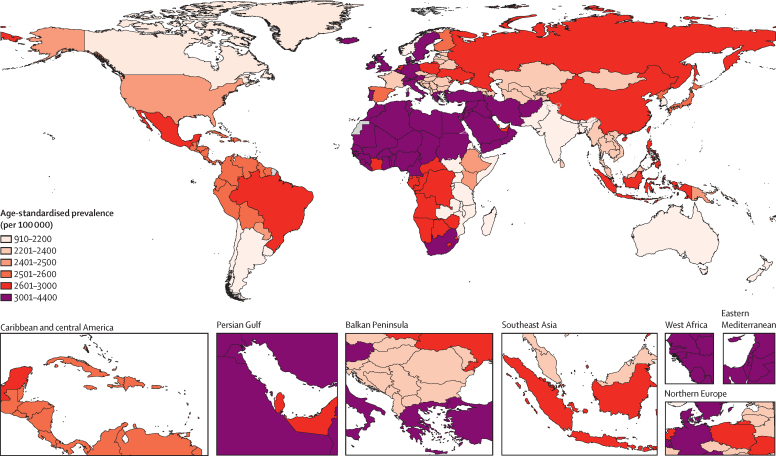
Age-standardised prevalence (per 100 000 population) of neck pain by location for male and female sexes combined, 2020

A total of 92 sources were used in the current analysis, spanning 27 countries and territories over 12 regions (appendix p 5). In 2020, there were an estimated 203 million (95% UI 163–253) people (all ages) with neck pain globally, representing an increase of 77·3% (70·1–84·9), from 115 million (91·6–142) in 1990 (table; appendix p 9). Between 1990 and 2020, the global age-standardised prevalence rate remained almost constant, at 2440 (1960–3030) per 100 000 population in 1990 and 2450 (1960–3040) per 100 000 in 2020 (figure 1; table). The percentage change in age-standardised rates of prevalence from 1990 to 2020 was 0·2% (–1·3 to 1·7).

**Table:** Prevalence, YLDs, age-standardised rates of prevalence and YLDs per 100 000 population in 2020, and percentage change between 1990 and 2020 for neck pain globally, by GBD super-region and region

		**Prevalence**				**YLDs**		
		Counts, 2020	Percentage change in counts, 1990–2020	Age-standardised rate per 100 000, 2020	Percentage change in age-standardised rate per 100 000, 1990–2020	Counts, 2020	Age-standardised rate per 100 000, 2020	Percentage change in age-standardised rate per 100 000, 1990–2020
Global		203 000 000 (163 000 000 to 253 000 000)	77·3% (70·1 to 84·9)	2450 (1960 to 3040)	0·2% (−1·3 to 1·7)	20 200 000 (13 700 000 to 28 800 000)	244·0 (165·0 to 346·0)	0·2% (−1·3 to 1·6)
	Males	81 600 000 (65 400 000 to 102 000 000)	72·3% (64·7 to 79·6)	2000 (1600 to 2480)	17·2% (15·6 to 18·7)	8 210 000 (5 510 000 to 11 800 000)	201·0 (135·0 to 286·0)	17·0% (15·0 to 19·7)
	Females	122 000 000 (97 900 000 to 152 000 000)	80·8% (72·2 to 89·3)	2890 (2330 to 3620)	13·6% (12·0 to 15·0)	12 000 000 (8 160 000 to 17 000 000)	286·0 (194·0 to 406·0)	13·2% (11·6 to 15·0)
Central Europe, eastern Europe, and central Asia		12 800 000 (10 300 000 to 15 900 000)	11·7% (8·2 to 15·6)	2540 (2040 to 3190)	−0·8% (−1·1 to −0·5)	1 260 000 (853 000 to 1 790 000)	254·0 (170·0 to 358·0)	−0·5% (−1·2 to 0·1)
	Central Asia	2 200 000 (1 720 000 to 2 780 000)	60·4% (53·3 to 66·1)	2340 (1860 to 2910)	−0·2% (−0·3 to −0·1)	221 000 (147 000 to 320 000)	234·0 (157·0 to 334·0)	−0·2% (−1·5 to 1·0)
	Central Europe	3 590 000 (2 900 000 to 4 440 000)	8·9% (4·0 to 14·2)	2450 (1960 to 3080)	0·3% (0·1 to 0·6)	355 000 (240 000 to 505 000)	246·0 (165·0 to 349·0)	0·7% (−0·1 to 1·5)
	Eastern Europe	6 970 000 (5 680 000 to 8 660 000)	3·2% (−0·1 to 7·2)	2670 (2150 to 3360)	−0·1% (−0·2 to 0·0)	687 000 (465 000 to 981 000)	266·0 (179·0 to 375·0)	0·1% (−0·7 to 0·8)
High income		35 200 000 (28 000 000 to 43 100 000)	28·0% (22·9 to 32·8)	2560 (2060 to 3170)	−3·8% (−5·1 to −2·7)	3 470 000 (2 360 000 to 4 730 000)	256·0 (170·0 to 357·0)	−4·0% (−5·5 to −2·7)
	Australasia	494 000 (399 000 to 612 000)	66·5% (59·5 to 73·8)	1360 (1070 to 1670)	0·0% (−0·1 to 0·1)	48 800 (33 100 to 66 700)	135·0 (90·4 to 188·0)	−0·0% (−3·6 to 2·6)
	High-income Asia Pacific	5 750 000 (4 650 000 to 7 100 000)	26·4% (18·3 to 36·4)	2260 (1810 to 2870)	−2·3% (−3·3 to −1·6)	573 000 (387 000 to 802 000)	229·0 (153·0 to 328·0)	−2·1% (−3·2 to −1·0)
	High-income North America	10 300 000 (8 310 000 to 12 700 000)	40·1% (33·0 to 46·9)	2380 (1880 to 3000)	−0·2% (−0·3 to −0·1)	1 010 000 (679 000 to 1 410 000)	235·0 (158·0 to 336·0)	−1·1% (−1·7 to −0·2)
	Southern Latin America	1 570 000 (1 250 000 to 1 990 000)	55·9% (51·4 to 59·4)	2100 (1680 to 2670)	−0·0% (−0·1 to −0·0)	157 000 (105 000 to 218 000)	210·0 (139·0 to 295·0)	−0·4% (−2·5 to 1·7)
	Western Europe	17 100 000 (13 800 000 to 21 200 000)	19·5% (14·9 to 24·0)	2970 (2370 to 3670)	−4·5% (−6·6 to −2·5)	1 680 000 (1 140 000 to 2 270 000)	297·0 (196·0 to 413·0)	−4·4% (−6·7 to −2·4)
Latin America and Caribbean		16 800 000 (13 400 000 to 21 100 000)	93·9% (82·1 to 105·0)	2690 (2150 to 3350)	0·1% (−0·0 to 0·2)	1 670 000 (1 140 000 to 2 400 000)	267·0 (182·0 to 380·0)	−0·0% (−0·6 to 0·5)
	Andean Latin America	1 590 000 (1 250 000 to 2 010 000)	112·0% (101·0 to 122·0)	2480 (1960 to 3110)	−0·0% (−0·1 to 0·1)	159 000 (106 000 to 228 000)	248·0 (166·0 to 353·0)	−0·3% (−2·4 to 1·3)
	Caribbean	1 260 000 (998 000 to 1 580 000)	60·0% (52·5 to 67·4)	2480 (1970 to 3120)	0·0% (−0·0 to 0·1)	125 000 (85 200 to 179 000)	247·0 (168·0 to 354·0)	−0·3% (−1·4 to 1·0)
	Central Latin America	6 980 000 (5 570 000 to 8 780 000)	101·0% (87·4 to 113·0)	2680 (2150 to 3350)	−0·0% (−0·2 to 0·1)	697 000 (470 000 to 1 000 000)	267·0 (181·0 to 381·0)	−0·0% (−0·8 to 0·8)
	Tropical Latin America	6 990 000 (5 540 000 to 8 740 000)	90·7% (78·8 to 103·0)	2790 (2220 to 3480)	0·1% (−0·0 to 0·2)	692 000 (470 000 to 989 000)	277·0 (188·0 to 392·0)	0·0% (−0·7 to 0·9)
North Africa andMiddle East		22 200 000 (17 500 000 to 28 400 000)	143·0% (132·0 to 151·0)	3750 (3010 to 4720)	−0·8% (−1·1 to −0·6)	2 210 000 (1 470 000 to 3 210 000)	370·0 (251·0 to 533·0)	−1·4% (−2·1 to −0·7)
South Asia		27 600 000 (22 200 000 to 34 600 000)	112·0% (105·0 to 118·0)	1590 (1290 to 1970)	0·4% (0·2 to 0·5)	2 740 000 (1 880 000 to 3 980 000)	157·0 (109·0 to 228·0)	0·9% (0·1 to 1·7)
Southeast Asia, east Asia, and Oceania		67 300 000 (54 400 000 to 83 600 000)	85·8% (69·1 to 102·0)	2520 (2010 to 3130)	2·1% (−2·5 to 5·8)	6 730 000 (4 500 000 to 9 560 000)	253·0 (170·0 to 357·0)	2·2% (−2·3 to 6·1)
	East Asia	49 900 000 (40 000 000 to 62 000 000)	80·5% (61·7 to 100·0)	2560 (2040 to 3170)	2·6% (−3·4 to 7·6)	4 980 000 (3 340 000 to 7 120 000)	257·0 (173·0 to 362·0)	2·7% (−3·0 to 7·9)
	Oceania	259 000 (209 000 to 328 000)	138·0% (132·0 to 143·0)	2430 (1990 to 3010)	0·1% (−0·0 to 0·1)	26 000 (17 500 to 37 300)	240·0 (164·0 to 335·0)	0·2% (−1·7 to 2·2)
	Southeast Asia	17 200 000 (13 600 000 to 21 900 000)	102·0% (91·0 to 112·0)	2400 (1920 to 3010)	0·1% (−0·2 to 0·4)	1 720 000 (1 140 000 to 2 490 000)	240·0 (161·0 to 341·0)	0·5% (−0·3 to 1·1)
Sub-Saharan Africa		21 500 000 (17 200 000 to 27 600 000)	145·0% (140·0 to 149·0)	2750 (2220 to 3490)	1·4% (0·1 to 2·6)	2 160 000 (1 430 000 to 3 150 000)	272·0 (183·0 to 395·0)	1·7% (0·3 to 2·8)
	Central sub-Saharan Africa	2 450 000 (1 950 000 to 3 140 000)	160·0% (157·0 to 163·0)	2580 (2090 to 3320)	−0·5% (−0·7 to −0·2)	244 000 (164 000 to 354 000)	254·0 (172·0 to 369·0)	0·1% (−1·9 to 2·1)
	Eastern sub-Saharan Africa	6 330 000 (5 020 000 to 8 020 000)	143·0% (140·0 to 146·0)	2220 (1790 to 2790)	0·1% (0·0 to 0·2)	636 000 (422 000 to 943 000)	220·0 (150·0 to 325·0)	0·7% (−0·2 to 1·5)
	Southern sub-Saharan Africa	2 150 000 (1 720 000 to 2 740 000)	88·2% (80·8 to 93·7)	2880 (2320 to 3640)	−0·0% (−0·3 to 0·2)	212 000 (142 000 to 314 000)	283·0 (192·0 to 417·0)	−0·9% (−1·7 to 0·1)
	Western sub-Saharan Africa	10 600 000 (8 370 000 to 13 600 000)	159·0% (152·0 to 166·0)	3240 (2610 to 4100)	3·0% (0·4 to 5·6)	1 070 000 (700 000 to 1 530 000)	321·0 (214·0 to 458·0)	3·4% (0·5 to 5·5)

Data in parentheses are 95% uncertainty intervals. GBD=Global Burden of Diseases, Injuries, and Risk Factors Study. YLDs=years lived with disability.

Total YLDs increased by 76·2% (95% UI 69.3–83·7), from 11·5 million (7·7–16·4) in 1990 to 20·2 million (13·7–28·8) in 2020 (table). The age-standardised YLD rate was 244 (165–346) per 100 000 in 2020, which was similar to the estimate of 243 (165–345) per 100 000 in 1990, a percentage change of 0·2% (–1·3 to 1·6).

**Figure 2:**
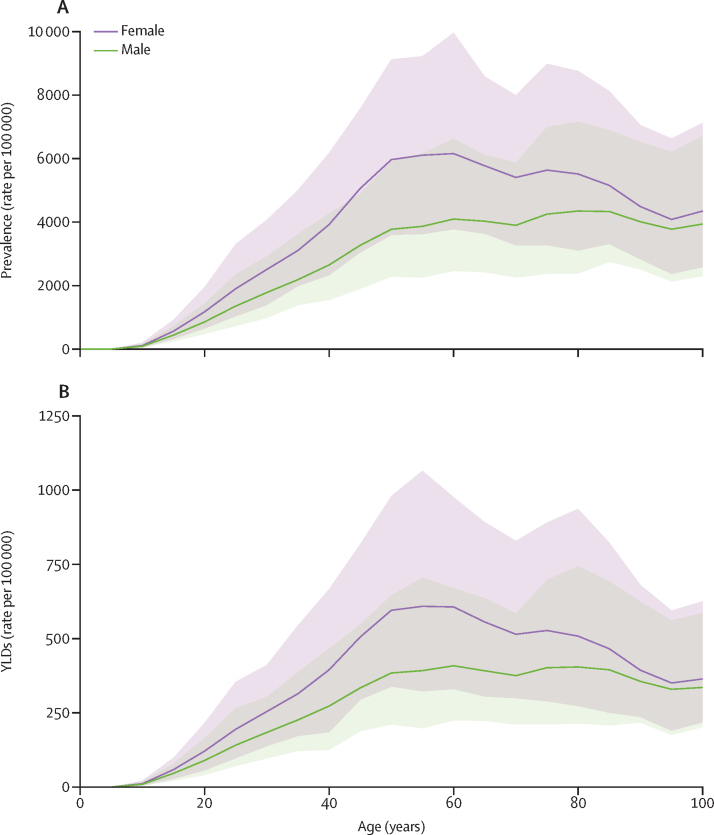
Global neck pain prevalence (A) and YLD (B) rate per 100 000 population by age and sex in 2020 The shaded area represents 95% uncertainty intervals, including overlap between male and female. YLD=years lived with disability.

Prevalence of neck pain was higher in females than males: the 2020 age-standardised prevalence was 2890 (95% UI 2330–3620) per 100 000 in females and 2000 (1600–2480) per 100 000 in males. Similarly, YLD rates were higher in females (286 [194–406] per 100 000) than males (201 [135–286] per 100 000). Prevalence and YLD rates peaked between the ages of 50 years and 74 years (figure 2).

Among the 21 GBD regions, the region with the highest age-standardised point prevalence of neck pain was north Africa and the Middle East (3750 [95% UI 3010–4720] per 100 000), and the region with the lowest was Australasia (1360 [1070–1670]; figure 1; table). Regional-level prevalence estimates of neck pain are provided in the table, with country-level prevalence reported in the appendix (p 9).

The GBD super-region of north Africa and the Middle East had the highest age-standardised YLD rates, at 370 (251–533) per 100 000. The lowest rates were in the south Asia super-region with an age-standardised YLD rate of 157 (109–228) per 100 000. From 1990 to 2020, changes in age-standardised YLDs ranged from a decrease of 4·0% (2·7–5·5) in the high-income super-region to a 2·2% (–2·3 to 6·1) increase in southeast Asia, east Asia, and Oceania super-region.

**Figure 3:**
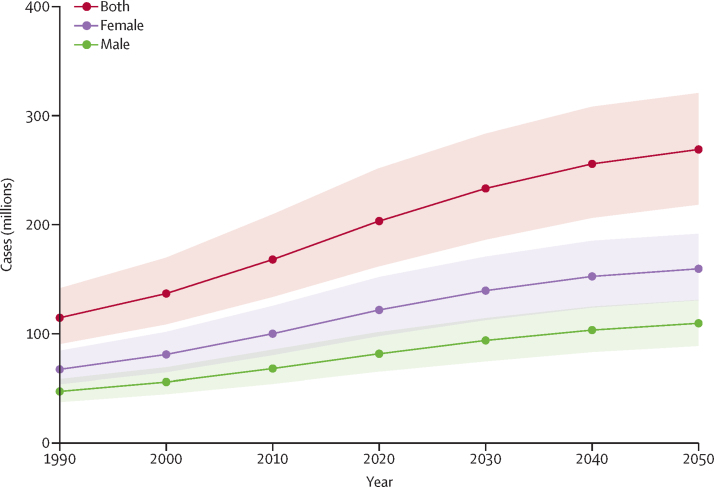
Estimated global cases of neck pain from 1990 to 2050, with 95% uncertainty intervals

Based on forecasted changes in population and prevalence estimates, in 2050 an estimated 269 million (219–322) people globally will have neck pain, an increase of 32·5% (23·9–42·3) from 2020 to 2050 (figure 3). Of the total neck pain cases in 2050, there are 160 million (131–192) forecasted cases in females, and 109 million (88·8–131) forecasted cases in males.

**Figure 4:**
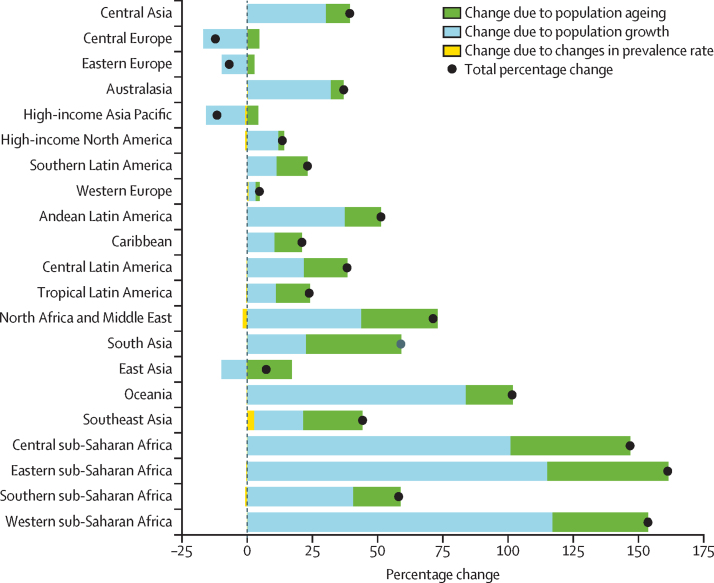
Decomposition analysis of forecasted change in neck pain prevalence by region, 2020 to 2050

Three regions with declining population growth—high-income Asia Pacific, central Europe, and eastern Europe—were forecasted to have a decrease in total neck pain cases between 2020 and 2050 (figure 4). The regions with a projected increase in neck pain of more than 100% included central sub-Saharan Africa, eastern sub-Saharan Africa, western sub-Saharan Africa, and Oceania (figure 4; appendix p 27).

Decomposition analysis shows the relative contribution of population ageing, population growth, and changes in prevalence to the forecasted increase in total cases of neck pain across regions (figure 4). In most regions, population growth is the largest contributor to projected prevalence changes. In sub-Saharan Africa and Oceania, for example, the population growth contributes 83% change in Oceania, and more than 100% change in central, eastern, and western sub-Saharan Africa. However, population growth is a negative contributor in regions within central Europe, eastern Europe, high-income Asia Pacific, and east Asia. Changes in prevalence have a minor effect on forecasts. Population ageing had a considerable contribution to the increased prevalence in the Latin American countries and south and east Asia.

## Discussion

The present study provides the most up-to-date and comprehensive prevalence and YLD estimates of neck pain at global and regional levels, including the addition of opportunistic studies from GBD collaborators adding to previous data. The forecasted prevalence to 2050 is also presented.

We found that the global age-standardised point prevalence and the YLD rate of neck pain have remained consistent over the past three decades but are forecasted to increase by more than 30% in the next three decades. Total YLDs increased by 78% from 1990 to 2020, suggesting that the increase is driven mainly by population growth and an ageing population. Our decomposition analysis found that, in most regions, population growth was the largest contributor to the forecasted increase in neck pain prevalence. Females had a higher prevalence of insidious or traumatic neck pain than males, and this pattern is expected to continue over the next three decades. Although multifactorial, geometric differences in the size of cervical vertebrae and paraspinal musculature, which are smaller in females than males,^21^ might contribute to the sex-dependent discrepancies in the prevalence of neck pain following trauma (eg, motor vehicle collision). Studies have suggested biological (genetic) factors might contribute to the sex disparities in perception of pain.^22^ The reasons for this sex disparity need to be explored.

GBD 2019 found neck pain ranked 11th out of the 369 conditions in terms of YLDs.^3^ Given this ongoing high global burden, with neck pain featuring in the leading causes of YLDs since 1990, it is disappointing that effective prevention and management interventions have not been forthcoming,^23^ and we still do not understand all the determinants and risk factors. Lack of leadership at country and global levels towards implementation of specific musculoskeletal health strategies has probably contributed to minimal prioritisation of musculoskeletal disorders among policy makers and thereby funders and primary health-care practitioners.^24^ Potential reasons are that neck pain is not a homogeneous condition or single disease but rather the symptom state associated with a broad range of conditions, and each person has their own unique experience with their pain. Causes of neck (and low back) pain might be unknown and have not been well defined in population estimates and might include muscle strain (including bad sleeping posture, repetitive movements or injury, carrying of heavy goods, and wearing of heavy loads in the neck region); inflammatory and degenerative changes in the intervertebral discs, joints, ligaments, and nerves; or a non-catastrophic injury from a motor vehicle collision, work-related or sports-related event, or degenerative condition (eg, secondary osteoarthritis of cervical facet joints or degenerative cervical myelopathy).^25^, ^26^ It is also possible the heterogeneity of the clinical course should be reconceptualised as both a tissue-based and stress-based interaction where individual diatheses influence the interaction between inherent vulnerability and environmental stressors. By endorsing the concept of diatheses, we encourage recognition that no two people will respond the same to their neck pain. Whether those responses are driven by the pain experience, genetics, microbiome, life experiences, cultural beliefs and pressures, or (more likely) some contribution from all of these, the challenge for clinicians and researchers is to work beyond their typical academic boundaries to find points of convergence that can start to unravel what continues to be a burdensome and recalcitrant problem: acute and chronic neck pain.^7^, ^27^, ^28^

It is of particular concern that both prevalence and YLDs for neck pain peak during the working years, between the ages of 45 years and 74 years. Neck pain has been reported to result in negative effects on employee productivity with considerable financial effects placed on the employee, employer, and health-care systems. A study in Spain^12^ reported that 12·3% of Spanish workers missed at least 7 days of work over the year due to neck pain. A study in Greece found that 8·6% of working individuals (aged 15–65 years) missed an average of 4·6 days of work over the course of 1 month due to neck pain.^13^ Neck pain might also be responsible for presenteeism, where those with neck pain stay at work but are not as productive.^29^ Tailored early exercise aimed at reducing muscular tension and workplace ergonomic interventions, such as keyboard position close to the body and varying work tasks,^11^ along with the addition of strengthening exercises at the workplace,^30^ have been suggested to relieve neck pain, increase productivity, and decrease time lost from work.^31^, ^32^ Evidence remains inconclusive as to whether such approaches are affecting the global burden of neck pain. We opine that there is a need to develop better causal explanations and identify more effective interventions for prevention and management to mitigate the neck pain suffered by individuals and the resulting disease burden.^7^, ^33^

With the exception of north Africa and the Middle East and south Asia, which had the highest and lowest rates, respectively, age-standardised rates of prevalence and YLDs were similar across the GBD super-regions. Cultural and lifestyle differences might come into play when examining regional differences. Regions where there is a greater reliance on neck transfer of heavy goods, particularly among women, might show higher prevalence and YLDs for neck pain. However, in high-income countries, high prevalence and YLDs might be attributed to a greater use of technological devices such as computers, laptops, and mobile phones. A more sedentary lifestyle and home working with poor ergonomics during the COVID-19 pandemic might have also contributed to greater neck pain, which are not captured in our estimates' or something similar. This raised a question as to whether it is a cause or effect of the greater availability and access to health practitioners, compensation insurance, diagnostic testing such as imaging, and subsequent treatment options, including spinal manipulation, spinal injections, or surgery, which might not always reflect current best evidence practice.^34^ The use of imaging and diagnostic tests might contribute to an increase in high-cost, low-value care and could be associated with poorer outcomes,^35^ including the possibility of producing iatrogenic neck pain, for which a solution challenges the acumen of even the most astute clinician.^36^, ^37^ Evidence should be provided to both clinicians and patients to ensure high-value care whereby informed treatments and adequate pain management options prevail. We would recommend that a focus for future research should involve an interdisciplinary collaborative effort among researchers, clinicians, patient groups, policy makers, and funding bodies, to optimise diagnostics and treatments through the global implementation of consistent research methodologies that can be replicated, including new discovery and innovation. Recommendations for value-based care for patients with chronic musculoskeletal pain could then be realised.^38^ In the absence of trauma and red flags, such as signs and symptoms of infection or upper motor neuron pathology or associated chest pain or shortness of breath, routine imaging and the associated radiation exposure should not be used in the vast majority of cases, and patients should be encouraged to be active and return to their own bespoke levels of normal functioning,^39^ whereby satisfaction and recovery can be measured and reported.^40^

Strengths of this analysis include the use of modelling techniques to adjust available data to enable estimates for all countries and regions through the use of regional priors and country-level covariates. The current analyses also include, for the first time, prevalence projections of neck pain to 2050.

A first limitation of the current GBD study is the lack of primary data, particularly from low-income countries, and as a result there is a high risk of compositional bias; additional prevalence and incidence data are crucial to improve the accuracy and reporting of results. Uncertainty in these estimates is based on 100 draws using the fractional interpolation method, and although we acknowledge that 1000 draws would be ideal, we would expect minimal bias in the results and uncertainty intervals from having used 100 draws only. There is a lack of data from low-income countries, where neck pain might not be reported and where more physical labour might be undertaken on a daily basis, such as neck transfer of heavy goods. We strongly encourage resources to be directed to collection of data within these low-income countries in addition to data from rural versus urban areas and differing ethnicity and education levels.

Moreover, the heterogeneity of neck pain data requires the usage of data adjustment methods that introduce additional uncertainty. It can be unstable due to limited input data, and potentially mask true differences in disease burden, too. There remains a need for collection of neck pain data in a standard way that they are generalisable worldwide. Validated musculoskeletal questionnaires such as Community Oriented Program for Control of Rheumatic Diseases (COPCORD) surveys and the Global Alliance for Musculoskeletal Health Musculoskeletal (G-MUSC) Survey Module hold the potential to standardise survey methodology and increase comparability.^41^, ^42^

A limitation to interpreting and therefore responding to the neck pain burden estimates is how it has been defined as pain that “lasted for at least one day”, meaning that chronicity cannot be fully appreciated. We would advise that chronic neck pain should be quantified separately from shorter-duration episodes of neck pain in future GBD iterations. Wider literature suggests about 20% of people with neck pain do not fully recover, with potential poor prognostic factors including greater pain intensity at onset, report of associated headache, higher levels of distress, and co-occurrence with low back pain. A focus on only reducing neck pain but not physical impairments in the articular and muscular system might be reasons for recurrent episodes. Delays in access to evidence-based treatments for pain might result in significant deterioration in health-related quality of life and psychological wellbeing,^2^ which then leads to chronic presentation across services.

To obtain estimates of neck pain severity to include in the modelling process, proportions of people with mild, moderate, or severe neck pain were derived from analysis of US MEPS data, and those proportions were attributed across all data. The GBD methodology assumes the same distribution of severity for all countries regardless of access to health care, and for males and females alike and across ages. It is not known how this affects prevalence estimates and severity distributions. Methods to take into account potential variation in severity across countries, between sexes, and across ages should be developed, in addition to prioritisation of better global data collection.

No data on risk factors for neck pain were included that could have assisted the modelling process. As a result, the model suffers from an absence of predictive covariates, which have been suggested by previous research, including genetics, psychopathology, smoking, obesity, sedentary lifestyles, trauma, back pain, and those within the workplace.^10^, ^43^ As evidence-based data on risk factors are required for inclusion in GBD models, further research on modifiable risk factors for neck pain is needed to strengthen estimates in data-sparse locations and provide relevant information for policy makers, as well as the patients themselves, regarding assessment and prevention. Furthermore, the forecasting decomposition analysis shows that there is little change in neck pain case numbers attributable to the age-standardised rate. Instead, it is wholly due to demographics across GBD regions. Additionally, our projections did not account for COVID-19's impact on the prevalence or burden of neck pain, such as decreased access to treatment.

From 1990 to 2020, a reduction in the burden of neck pain was not realised, and based on the forecast to 2050 absolute burden will increase significantly. Similar to the burden of other non-communicable diseases, we suggest that interdisciplinary and collaborative action that includes the affected patient groups at the primary care level is needed, including physical exercises, physical therapy, and advice to self-manage where appropriate, if we are to realise a reduction in the burden of neck pain.^44^ To enable more accurate estimation of prevalence and burden over time and across geographies, consensus on a standard definition that allows more targeted models of service delivery and referral, in addition to collecting comparable data on neck pain and its risk factors are needed. Such data would enable health systems to deliver high-value musculoskeletal care based on a global framework of action.^38^ This evidence is a call to action for policy makers to strengthen the capacity of their health systems to deliver quality musculoskeletal care services, including early detection, management, and long-term care; undertake research that incorporates sex and age as biological variables influencing the differential burdens and consequences of neck pain; and invest adequate resources to prevent neck pain such as improve the working environment of their citizens using ergonomics.

The global age-standardised prevalence and YLD rate of neck pain have remained constant over the past three decades. Females and middle-aged and older populations continue to have the highest burden of neck pain, the latter of which represents a non-modifiable factor. The current state of the prevalence and disability associated with neck pain needs to be highlighted and combined with greater patient and consumer group engagement to drive real-world policy at country and global leadership levels towards lessening its burden across an ageing and culturally diverse world. Meanwhile, the global musculoskeletal community needs to generate better country-based estimates, using standardised definitions, addressing modifiable risk factors and covariates so that trends in prevalence and severity over time can be documented. Future discovery and innovation towards identifying and preventing the transition from acute to chronic neck pain with patient-centred interventions is the next step.

For more on the **Global Health Data Exchange** see http://ghdx.healthdata.org/gbd-2019/data-input-sources

## Data sharing

The findings of this study are supported by data available in public online repositories, data publicly available upon request of the data provider, and data not publicly available due to restrictions by the data provider. Non-publicly available data were used under licence for the current study but can be made available by the authors upon reasonable request and with permission of the data provider. Data sources used in this analysis are listed in the appendix (pp 28–33).

## Declaration of interests

B Antony reports grants from the Rebecca Cooper Foundation and the Nat Rem Ltd grant; payment or honoraria for lectures, presentations, speakers bureaus, or educational events from Nat Red Ltd and IRACON; outside the submitted work. A M Briggs reports grants or contracts paid to his institution from the Bone and Joint Decade Foundation, AO Alliance, Canadian Memorial Chiropractic College, Australian Rheumatology Association, Pan-American League of Associations for Rheumatology, World Federation of Chiropractic, and Asia Pacific League of Associations for Rheumatology; consulting fees from WHO; payment or honoraria for lectures, presentations, speakers bureaus, manuscript writing or educational events from the American College of Rheumatology; support for attending meetings/travel from WHO; outside the submitted work. R Buchbinder reports grants or contracts from Australian National Health and Medical Research Council (NHMRC), Australian Commonwealth government, HCF Foundation, Cabrini Foundation, and Arthritis Australia; royalties from UptoDate for plantar fasciitis; outside the submitted work. J M Elliott reports other financial support from Orofacial Therapeutics LLC, outside the submitted work. R C Franklin reports grants or contracts from Heatwaves in Queensland – Queensland Government, Arc Flash –Human Factors – Queensland Government, and Mobile Plant Safety – Agrifutures; honoraria for lectures, presentations, speakers bureaus, manuscript writing or educational events from the World Safety Conference 2022 - Conference Convener; support for attending meetings and/or travel from ACTM – Tropical Medicine and Travel Medicine Conference 2022 and ISTM – Travel Medicine Conference, Basel 2023; leadership or fiduciary roles in board, society, committee or advocacy groups, paid or unpaid with Kidsafe as a Director, Auschem as a Director, the International Society for Agricultural Safety and Health (ISASH) on the Governance Committee, Farmsafe as a Director, and the Public Health Association of Australia (PHAA) as the Injury Prevention SIG Convenor; outside the submitted work. J J Hebert reports financial support from the New Brunswick Health Research Foundation and the Canadian Chiropractic Research Foundation; outside the submitted work. D Kopansky-Giles reports leadership roles in board, society, committee or advocacy groups, paid or unpaid with the Global Alliance for Musculoskeletal Health as a member of the executive committee; outside the submitted work. K Krishan reports other non-financial support from UGC Centre of Advanced Study, CAS II, Department of Anthropology, Panjab University, Chandigarh, India; outside the submitted work. E Mathews reports grants or contracts from Wellcome DBT India Alliance; outside the submitted work. S Sharma reports grants or contracts from the International Association for the Study of Pain John J Bonica Postdoctoral Fellowship (the funder does not have any influence on S Sharma's research); support for attending meetings/travel from the International Association for the Study of Pain (IASP) Congress in Toronto in 2022; other nonfinancial support as a Board Member of the IASP Pain Mind and Movement Special Interest Group of the IASP, Global Year Task Force Member 2022 and 2023, and as a Board Member of Global Alliance of Partners for Pain Advocacy; outside the submitted work. J A Singh reports consulting fees from Crealta/Horizon, Medisys, Fidia, PK Med, Two labs Inc., Adept Field Solutions, Clinical Care options, Clearview healthcare partners, Putnam associates, Focus forward, Navigant consulting, Spherix, MedIQ, Jupiter Life Science, UBM LLC, Trio Health, Medscape, WebMD, and Practice Point communications; and the National Institutes of Health and the American College of Rheumatology; payment or honoraria for lectures, presentations, speakers bureaus, manuscript writing or educational events from the speaker's bureau of Simply Speaking; support for attending meetings and/or travel from OMERACT as a member of the steering committee; participation on a Data Safety Monitoring Board or Advisory Board with the FDA Arthritis Advisory Committee; leadership or fiduciary roles in board, society, committee or advocacy groups, paid or unpaid as a past steering committee member of the OMERACT, an international organization that develops measures for clinical trials and receives arms length funding from 12 pharmaceutical companies, Co-Chair of the Veterans Affairs Rheumatology Field Advisory Committee, and the editor and Director of the UAB Cochrane Musculoskeletal Group Satellite Center on Network Meta-analysis; stock or stock options in Atai Life Sciences, Kintara Therapeutics, Intelligent Biosolutions, Acumen Pharmaceutical, TPT Global Tech, Vaxart Pharmaceuticals, Atyu Biopharma, Adaptimmune Therapeutics, GeoVax Labs, Pieris Pharmaceuticals, Enzolytics Inc., Seres Therapeutics, Tonix Pharmaceuticals Holding Corp., and Charlotte's Web Holdings, Inc, as well as previously owned stock options in Amarin, Viking and Moderna Pharmaceuticals; outside the submitted work. D M Walton reports grants from Natural Science and Engineering Research Council (NSERC) in Canada; Consulting fees for digital MSK health start-up; payment for expert testimony from the College of Physiotherapists of Ontario; patents planned, issued, or pending for a panel of blood markers to detect ‘risk’ of chronic pain after MSK trauma – including but not specific to the neck; stock or stock options in TSX stocks; other financial support as a co-author of a book on musculoskeletal pain from Handspring Publishers; all outside the submitted work. A Woolf reports leadership or fiduciary roles in board, society, committee or advocacy groups, paid or unpaid with the Global Alliance for Musculoskeletal Health as a co-chair; outside the submitted work.
