# Supplementary material for: Global, regional, and national burden of neck pain, 1990–2020, and projections to 2050: a systematic analysis of the Global Burden of Disease Study 2021
Source: Lancet Rheumatol. 2024 Feb 19;6(3):e142–55. doi: 10.1016/S2665-9913(23)00321-1 (PMC10897950; doi:10.1016/S2665-9913(23)00321-1)
Supplement: Supplementary appendix [file mmc1.pdf]

THE LANCET
Rheumatology

Supplementary appendix

This appendix formed part of the original submission and has been peer reviewed. We post it as supplied by the authors.

Supplement to: GBD 2021 Neck Pain Collaborators. Global, regional, and national burden of neck pain, 1990–2020, and projections to 2050: a systematic analysis of the Global Burden of Disease Study 2021. *Lancet Rheumatol* 2024; **6:** e141–154.

Appendix

Supplementary material for Global, regional, and national burden of neck pain, 1990 to 2020 and projections to 2050: a systematic analysis of the Global Burden of Disease Study 2021

CONTENTS

	Page
**METHODS**	**1**
**Section 1: Statement of GATHER compliance**	**2**
**Section 2: Data seeking**	**3**
Supplemental Figure 1: PRISMA diagram of neck pain systematic review update from 2017–2020	4
Supplemental Figure 2: Distribution of data sources for neck pain	5
Supplemental Table 1. Super-regional, regional and national breakdown of locations with data sources for neck pain	5
Supplemental Table 2. National sources in countries with subnational estimation (From (http://ghdx.healthdata.org/gbd-2019/data-input-sources)	6
**Section 3. Sex-splitting, crosswalking and other data adjustment methods**	**6**
Supplemental Table 3: MR-BRT crosswalk adjustment factors for neck pain	7
**Section 4. Severity Distribution estimation**	**7**
Supplemental Table 4. Severity distribution, details on the severity levels for NP in GBD 2021 and the associated disability weight (DW) with that severity	8
**Section 5. Validation of forecasting model**	**8**
**Section 6. Results**	**9**
Supplemental Table 5: Prevalence, years lived with disability, age-standardised rates of prevalence, and YLDs per 100,000 in 2020 and percentage change between 1990 and 2020 for neck pain globally and by region and country	9
Supplemental Table 6. Forecast of neck pain age-standardised prevalence (%) and total cases (in millions) globally and by region, both sexes, from 2020 to 2050, with 95% uncertainty intervals	25
Supplemental Figure 3: Forecast to 2050 of neck pain age-standardised prevalence for GBD regions	27
List of data sources for Neck Pain estimates	**28**
Author contributions	**34**

Methods

This appendix provides supplemental figures and more detailed results for "Global, regional, and national burden of neck pain 1990–2020 and projections to 2050: a systematic analysis of the Global Burden of Disease Study 2021". Portions of this appendix have been reproduced or adapted from Vos et al. References are provided for reproduced sections.

Vos T, Lim SS, Abbafati C, et al. Global burden of 369 diseases and injuries in 204 countries and territories, 1990–2019: a systematic analysis for the Global Burden of Disease Study 2019, The Lancet, Volume 396, Issue 10258, 2020

Pages 1204-1222, **Section 1.** Statement of GATHER Compliance

This study complies with the Guidelines for Accurate and Transparent Health Estimates Reporting (GATHER) recommendations.

Checklist of information that should be included in new reports of global health estimates

**Item #**	**Checklist item**	**Reported on page #**
**Objectives and funding**		
**1**	Define the indicator(s), populations (including age, sex, and geographic entities), and time period(s) for which estimates were made.	P 6
**2**	List the funding sources for the work.	P. 4
**Data Inputs**		
For all data inputs from multiple sources that are synthesized as part of the study:		
**3**	Describe how the data were identified and how the data were accessed.	P. 6
**4**	Specify the inclusion and exclusion criteria. Identify all ad-hoc exclusions.	Appendix
**5**	Provide information on all included data sources and their main characteristics. For each data source used, report reference information or contact name/institution, population represented, data collection method, year(s) of data collection, sex and age range, diagnostic criteria or measurement method, and sample size, as relevant.	P. 6
**6**	Identify and describe any categories of input data that have potentially important biases (e.g., based on characteristics listed in item 5).	P. 7
For data inputs that contribute to the analysis but were not synthesized as part of the study:		
**7**	Describe and give sources for any other data inputs.	P. 8 and Appendix

For all data inputs:		
**8**	Provide all data inputs in a file format from which data can be efficiently extracted (e.g., a spreadsheet rather than a PDF), including all relevant meta-data listed in item 5. For any data inputs that cannot be shared because of ethical or legal reasons, such as third-party ownership, provide a contact name or the name of the institution that retains the right to the data.	P. 8 and Appendix
**Data analysis**		
**9**	Provide a conceptual overview of the data analysis method. A diagram may be helpful.	P.8 Appendix
**10**	Provide a detailed description of all steps of the analysis, including mathematical formulae. This description should cover, as relevant, data cleaning, data pre-processing, data adjustments and weighting of data sources, and mathematical or statistical model(s).	P.8
**11**	Describe how candidate models were evaluated and how the final model(s) were selected.	P.8
**12**	Provide the results of an evaluation of model performance, if done, as well as the results of any relevant sensitivity analysis.	P.8
**13**	Describe methods for calculating uncertainty of the estimates. State which sources of uncertainty were, and were not, accounted for in the uncertainty analysis.	P.8
**14**	State how analytic or statistical source code used to generate estimates can be accessed.	P.8
**Results and Discussion**		
**15**	Provide published estimates in a file format from which data can be efficiently extracted.	P.9
**16**	Report a quantitative measure of the uncertainty of the estimates (e.g. uncertainty intervals).	P.9-11
**17**	Interpret results in light of existing evidence. If updating a previous set of estimates, describe the reasons for changes in estimates.	P.12-13
**18**	Discuss limitations of the estimates. Include a discussion of any modelling assumptions or data limitations that affect interpretation of the estimates.	P.14

This checklist should be used in conjunction with the GATHER statement and Explanation and Elaboration document, found on gather-statement.org

Section 2. Data-seeking [1]

For GBD 2017, Ovid MEDLINE, EMBASE, CINAHL, CAB abstracts, WHOLIS, and SIGLE databases were searched for GBD 2010 and PUBMED was searched through October 2017, and these data were used for this update. There were no age, sex, or language restrictions. The terms neck pain, neck ache, neckache, and cervical pain individually and combined with each of the following terms: prevalen*, inciden*, cross-sectional, cross sectional, epidemiol*, survey, population-based, population based, population study, population sample.

Exclusion criteria were:

1. Sub-populations clearly not representative of the national population
2. Not a population-based study
3. Studies on a specific type of neck pain (eg, following neck fracture)
4. Low sample size (less than 150)

5. Review rather than original studies

Medical claims data from Taiwan (province of China) were additionally included. Taiwan claims data are from the Ministry of Health and Welfare (Taiwan) Taiwan National Health Insurance Claims Data 2016, and the case definition was based on ICD-10 codes. The ICD-10 code for neck pain is M54.2, while the ICD-9 code is 723.1.

A full list of data sources utilised in neck pain modelling for GBD 2021 is found here: http://ghdx.healthdata.org/gbd-2019/data-input-sources/ and are listed at the end of this Appendix.

Supplemental Figure 1: PRISMA diagram of neck pain systematic review update from 2017 to 2020

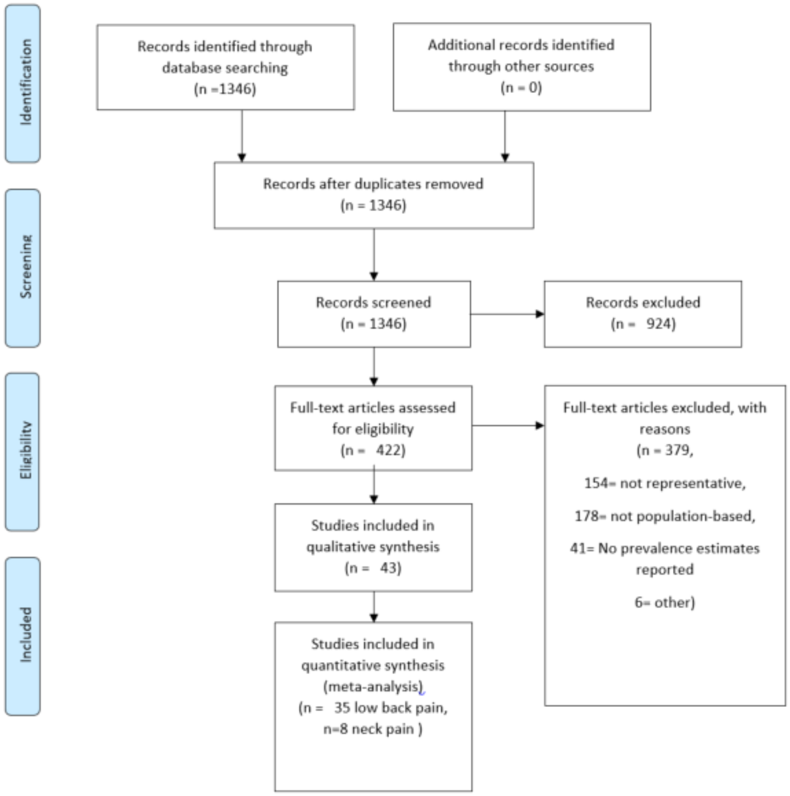

Note: One search was conducted for both neck pain and low back pain as data sources often included both conditions

Supplemental Figure 2: Distribution of data sources for neck pain

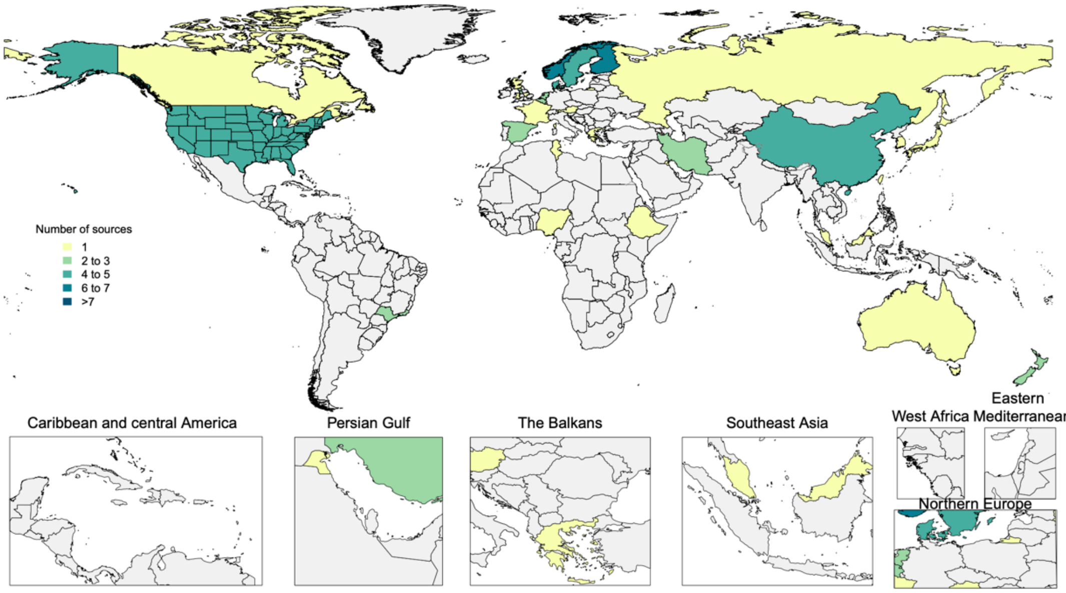

Supplemental Table 1. Super-regional, regional, and national breakdown of locations with data sources for neck pain

Super-region	Region	Countries
Central Europe, eastern Europe, and central Asia	Central Europe	Bosnia and Herzegovina
	Eastern Europe	Russia
High income	Australasia	New Zealand
	High income Asia Pacific	South Korea
	High income North America	USA
	Western Europe	Belgium, Denmark, England, Finland, France, Greece, Ireland, Netherlands, Norway, Scotland, Spain, Sweden, Wales
Latin America and Caribbean	Tropical Latin America	Brazil
North Africa and Middle East	North Africa and Middle East	Iran, Kuwait
Southeast Asia, east Asia, and Oceania	Southeast Asia	Malaysia
	East Asia	China, Taiwan (province of China)
	Oceania	Solomon Islands, Tonga
Sub-Saharan Africa	Western sub-Saharan Africa	Nigeria

Supplemental Table 2. National sources in countries with subnational estimation (From (http://ghdx.healthdata.org/gbd-2021/data-input-sources)

Location	National source counts	Subnational source counts	Total source counts
Brazil	0	5	5
China	0	4	4
UK	5	3	10
India	0	4	4
Iran	2	1	3
Japan	1	0	1
Nigeria	0	1	1
Norway	7	0	7
New Zealand	0	2	2
Russia	0	1	1
Sweden	0	4	4
USA	12	205	217

Section 3. Sex-splitting, crosswalking, and other data adjustment methods

Reported estimates of prevalence were split by age and sex where possible. If studies contained both sex-specific and separate age-specific estimates, the male-to-female ratio was used to proportionally split age-specific data and produce age- and sex-specific prevalence data. For studies with only "both"-sex data, studies with both male and female data were used to produce male/female ratios which were log-transformed and used as input data into a regression analysis using the MR-BRT (meta-regression—Bayesian, regularised, trimmed) tool. Model results were used to proportionally split "both"-sex data. Data with age ranges spanning more than 25 years were split into five-year age bins using age patterns derived from GBD 2021 final models for low back pain.

Supplemental Table 3: MR-BRT crosswalk adjustment factors for neck pain [1]

Data input	Reference or alternative case definition	Gamma	Beta coefficient, logit (95% CI)	Adjustment factor*
Point prevalence	Ref	0.30	---	---
Anatomical region too broad	Alt		0.97 (0.76 to 1.18)	2.63 (2.13 to 3.25)
Episode duration ≥3 months	Alt		–0.78 (–0.91 to –0.65)	0.46 (0.40 to 0.52)
Recall periods of 1 week to 1 month	Alt		1.13 (1.08 to 1.19)	3.10 (2.94 to 3.29)
Recall periods between 2 months and one year	Alt		1.68 (1.63 to 1.73)	5.37 (5.10 to 5.64)
Studies among schoolchildren	Alt		1.07 (0.78 to 1.36)	2.92 (2.18 to 3.90)
Activity-limiting neck pain	Alt		–1.13 (–1.14 to –1.12)	0.32 (0.32 to 0.33)

**MR-BRT crosswalk adjustments can be interpreted as the factor the alternative case definition is adjusted by to reflect what it would have been had it been measured using the reference case definition. If the log/logit beta coefficient is negative, then the alternative is adjusted up to the reference. If the log/logit beta coefficient is positive, then the alternative is adjusted down to the reference. The adjustment factors reflect the ratio of the alternative over the reference case definition. Thus, data for 'anatomical region too broad' are divided by 2.63.*

***The adjustment factor column is the exponentiated beta coefficient. For log beta coefficients, this is the relative rate between the two case definitions. For logit beta coefficients, this is the relative odds between the two case definitions.*

Section 4. Severity distribution information [1]

The severity distributions were derived from an analysis of the Medical Expenditure Panel Surveys (MEPS) in the USA. MEPS is an overlapping continuous panel survey of the USA non-institutionalised population whose primary purpose is to collect information on the use and cost of health care. Panels are two years long and are conducted in five rounds, which are conducted every five to six months. A new panel begins annually, while the last panel is in its second year. Each panel typically contains about 30,000 to 35,000 individual respondents (http://www.meps.ahrq.gov/survey_comp/hc_data_collection.jsp).

MEPS was initiated in 1996 but only began collecting health status data in the form of SF-12 responses in 2000. For GBD 2021 we used data from 2000–2014. Respondents self-administer the SF-12 twice per panel, at rounds two and four, typically about a year apart. Only adults 18 years and older completed the SF-12. MEPS also usually collects information on diagnoses based on self-report of reasons for encounters with health services. In addition, diagnoses are derived through additional questions on "problems that bother you" or conditions that led to "disability

days," ie, days out of role due to illness. Professional coders translate the verbatim text into three-digit ICD-9 codes. The main reason for neck pain being measured in MEPS relates to health care contact.

Supplemental Table 4. Severity distribution, details on the severity levels for NP in GBD 2021 and the associated disability weight (DW) with that severity. [1]

Severity level	Lay description	DW (95% CI)	Proportions
Neck pain, mild	This person has neck pain, and has difficulty turning the head and lifting things	0.052 (0.036 to 0.074)	0.67 (0.57 to 0.75)
Neck pain, moderate	This person has constant neck pain, and has difficulty turning the head, holding arms up, and lifting things	0.112 (0.079 to 0.162)	0.12 (0.08 to 0.19)
Neck pain, severe	This person has severe neck pain, and difficulty turning the head and lifting things. The person gets headaches and arm pain, sleeps poorly, and feels tired and worried	0.226 (0.147 to 0.323)	0.06 (0.05 to 0.07)
Neck pain, most severe	This person has constant neck pain and arm pain, and difficulty turning the head, holding arms up, and lifting things. The person gets headaches, sleeps poorly, and feels tired and worried	0.300 (0.199 to 0.434)	0.15 (0.11 to 0.20)

Section 5: Validation of forecasting model

Validation testing was conducted using estimates for osteoarthritis (OA) from 1990 to 2010 to project prevalence from 2010 to 2019 by age, sex, location, and year. The projections were then compared to the GBD OA prevalence results for this period by calculating the root mean squared error (RMSE) and bias (calculated as the median value of all predicted minus observed values by age, sex, location and year). Four tests were conducted: OA hip, OA knee, OA hand and OA other sites. In all the four tests the model RMSE was <0.0001.

References

1. Vos T, Lim SS, Abbafati C, Abbas KM, Abbasi M, Abbasifard M, et al. Global burden of 369 diseases and injuries in 204 countries and territories, 1990–2019: a systematic analysis for the Global Burden of Disease Study 2019. The Lancet. 2020; **396**(10258): 1204-22.

Section 6. Results

Supplemental Table 5: Prevalence, years lived with disability (YLDs), age-standardised rates of prevalence, and YLDs per 100,000 in 2020, and percentage change between 1990 and 2020 for neck pain globally, and by super-region, region, and country

Location	Number of prevalent cases (95% UI)	% change in number of prevalent cases from 1990 to 2020 (95% UI)	Age-standardised prevalence rate per 100 000 (95% UI)	% change in age-standardised prevalence rate from 1990 to 2020 (95% UI)	Number of YLDs (95% UI)	Age-standardised rate of YLDs per 100 000 in 2020 (95% UI)	% change in age-standardised rate of YLDs per 100 000 from 1990 to 2020 (95% UI)
GLOBAL	203 000 000 (163 000 000–253 000 000)	77.3 (70.1–84.9)	2 450 (1 960–3 040)	0·2 (-1·3–1·7)	20 200 000 (13 700 000–28 800 000)	244·0 (165·0–346·0)	0·2 (-1·3–1·6)
CENTRAL EUROPE, EASTERN EUROPE, AND CENTRAL ASIA	12 800 000 (10 300 000–15 900 000)	11.7 (8.2–15.6)	2 540 (2 040–3 190)	-0·8 (-1·1–-0·5)	1 260 000 (853 000–1 790 000)	254·0 (170·0–358·0)	-0·5 (-1·2–0·1)
**Central Asia**	2 200 000 (1 720 000–2 780 000)	60.4 (53.3–66.1)	2 340 (1 860–2 910)	-0·2 (-0·3–-0·1)	221 000 (147 000–320 000)	234·0 (157·0–334·0)	-0·2 (-1·5–1·0)
Armenia	82 800 (66 200–103 000)	9.7 (3.9–16.2)	2 340 (1 860–2 910)	0·0 (-0·1–0·1)	8 270 (5 590–11 900)	235·0 (158·0–335·0)	0·2 (-2·9–2·9)
Azerbaijan	268 000 (209 000–341 000)	78.8 (67.4–87.8)	2 330 (1 850–2 900)	-0·4 (-0·6–-0·2)	27 100 (18 100–39 700)	235·0 (157·0–339·0)	-0·4 (-2·7–2·3)
Georgia	102 000 (81 700–126 000)	-26.2 (-28.8–-22.9)	2 340 (1 860–2 910)	-0·4 (-0·5–-0·2)	10 100 (6 880–14 300)	234·0 (156·0–331·0)	-0·8 (-3·9–2·3)
Kazakhstan	451 000 (356 000–567 000)	26.9 (22.2–31.1)	2 340 (1 860–2 910)	-0·2 (-0·3–-0·0)	45 300 (30 100–65 800)	235·0 (155·0–336·0)	0·1 (-2·7–2·7)
Kyrgyzstan	144 000 (113 000–182 000)	68.3 (62.5–73.5)	2 340 (1 860–2 910)	-0·2 (-0·3–-0·0)	14 500 (9 690–20 800)	235·0 (158·0–335·0)	0·1 (-2·4–3·2)
Mongolia	79 600 (62 400–101 000)	115.0 (95.7–129.0)	2 340 (1 860–2 910)	0·3 (0·1–0·5)	8 040 (5 330–11 700)	235·0 (158·0–331·0)	0·5 (-2·2–4·0)

Tajikistan	198 000<br>(153 000–251 000)	117.0<br>(105.0–125.0)	2 330<br>(1 850–2 900)	-0·2<br>(-0·4–0·0)	20 000<br>(12 900–28 800)	234·0<br>(154·0–334·0)	-0·3<br>(-2·5–2·8)
Turkmenistan	117 000<br>(91 600–148 000)	77.8<br>(66.1–87.8)	2 330<br>(1 850–2 900)	-0·5<br>(-0·7–-0·2)	11 800<br>(7 720–17 000)	234·0<br>(155·0–333·0)	-0·5<br>(-3·0–2·4)
Uzbekistan	755 000<br>(589 000–963 000)	108.0<br>(94.4–118.0)	2 330<br>(1 860–2 910)	-0·1<br>(-0·3–0·0)	76 200<br>(49 800–110 000)	234·0<br>(157·0–333·0)	-0·4<br>(-2·9–2·6)
**Central Europe**	3 590 000<br>(2 900 000–4 440 000)	8.9<br>(4.0–14.2)	2 450<br>(1 960–3 080)	0·3<br>(0·1–0·6)	355 000<br>(240 000–505 000)	246·0<br>(165·0–349·0)	0·7<br>(-0·1–1·5)
Albania	76 500<br>(60 600–94 700)	14.8<br>(4.7–25.9)	2 330<br>(1 850–2 900)	0·4<br>(0·1–0·8)	7 610<br>(5 020–10 800)	233·0<br>(153·0–336·0)	0·3<br>(-2·9–3·2)
Bosnia and Herzegovina	98 500<br>(78 700–122 000)	-10.9<br>(-16.8–-4.0)	2 330<br>(1 850–2 900)	-0·1<br>(-0·2–0·1)	9 750<br>(6 480–13 800)	233·0<br>(155·0–331·0)	0·0<br>(-2·6–3·0)
Bulgaria	212 000<br>(170 000–264 000)	-10.7<br>(-14.7–-6.4)	2 330<br>(1 850–2 900)	-0·1<br>(-0·2–0·0)	20 900<br>(14 200–29 500)	233·0<br>(154·0–330·0)	0·1<br>(-2·3–2·8)
Croatia	129 000<br>(103 000–159 000)	-2.9<br>(-7.4–2.0)	2 330<br>(1 850–2 900)	-0·3<br>(-0·4–-0·1)	12 700<br>(8 570–17 900)	233·0<br>(157·0–332·0)	-0·2<br>(-3·0–2·4)
Czechia	321 000<br>(259 000–398 000)	17.1<br>(11.6–23.1)	2 320<br>(1 850–2 900)	-0·4<br>(-0·6–-0·2)	31 700<br>(21 800–44 600)	233·0<br>(156·0–334·0)	-0·2<br>(-2·8–2·5)
Hungary	296 000<br>(239 000–365 000)	4.5<br>(0.4–8.7)	2 330<br>(1 850–2 900)	-0·2<br>(-0·4–-0·1)	29 200<br>(19 700–41 100)	234·0<br>(156·0–335·0)	0·3<br>(-2·5–3·1)
Montenegro	17 700<br>(14 100–21 900)	17.1<br>(11.6–23.0)	2 330<br>(1 850–2 900)	-0·1<br>(-0·2–-0·0)	1 760<br>(1 170–2 500)	234·0<br>(155·0–335·0)	-0·2<br>(-3·0–2·4)
North Macedonia	62 400<br>(49 800–77 900)	30.5<br>(24.5–36.6)	2 320<br>(1 850–2 890)	-0·2<br>(-0·4–-0·1)	6 200<br>(4 160–9 060)	232·0<br>(156·0–335·0)	-0·3<br>(-3·6–2·7)
Poland	1 320 000<br>(1 080 000–1 640 000)	19.9<br>(14.5–25.8)	2 700<br>(2 170–3 380)	-0·2<br>(-0·3–-0·1)	131 000<br>(88 900–187 000)	270·0<br>(182·0–380·0)	0·4<br>(-0·6–1·4)
Romania	576 000<br>(463 000–709 000)	-3.4<br>(-8.7–2.2)	2 330<br>(1 850–2 900)	-0·2<br>(-0·2–-0·1)	57 000<br>(38 000–79 600)	234·0<br>(154·0–333·0)	0·4<br>(-3·2–3·5)
Serbia	253 000<br>(204 000–312 000)	5.9<br>(1.8–10.8)	2 330<br>(1 850–2 900)	-0·2<br>(-0·2–-0·1)	25 100<br>(16 800–35 000)	233·0<br>(155·0–333·0)	-0·2<br>(-3·2–2·3)

Slovakia	162 000 (131 000–200 000)	23.7 (17.7–29.0)	2 330 (1 850–2 900)	-0·3 (-0·5–-0·1)	16 100 (10 900–22 900)	234·0 (158·0–333·0)	0·4 (-2·5–3·0)
Slovenia	63 400 (50 500–78 100)	21.2 (15.2–28.3)	2 320 (1 850–2 890)	-0·6 (-0·9–-0·2)	6 250 (4 280–8 790)	233·0 (155·0–333·0)	-0·1 (-2·9–2·7)
**Eastern Europe**	6 970 000 (5 680 000–8 660 000)	3.2 (-0.1–7.2)	2 670 (2 150–3 360)	-0·1 (-0·2–0·0)	687 000 (465 000–981 000)	266·0 (179·0–375·0)	0·1 (-0·7–0·8)
Belarus	279 000 (223 000–345 000)	2.6 (-1.3–6.4)	2 340 (1 860–2 910)	-0·2 (-0·3–-0·1)	27 600 (18 600–39 100)	234·0 (155·0–335·0)	-0·0 (-2·6–3·8)
Estonia	39 100 (31 300–48 000)	-6.1 (-9.5–-1.6)	2 330 (1 860–2 900)	-0·6 (-0·8–-0·3)	3 850 (2 640–5 380)	234·0 (156·0–333·0)	-0·0 (-2·5–2·9)
Latvia	57 600 (45 800–70 900)	-19.6 (-22.9–-15.5)	2 340 (1 860–2 910)	-0·4 (-0·5–-0·2)	5 670 (3 840–7 980)	234·0 (156·0–335·0)	0·3 (-2·3–2·9)
Lithuania	84 400 (67 400–104 000)	-11.5 (-16.1–-6.9)	2 340 (1 860–2 910)	-0·2 (-0·3–-0·1)	8 310 (5 580–11 600)	234·0 (155·0–333·0)	0·3 (-2·3–3·0)
Republic of Moldova	109 000 (87 000–135 000)	1.6 (-2.7–6.3)	2 340 (1 860–2 910)	-0·3 (-0·4–-0·2)	10 800 (7 210–15 400)	234·0 (156·0–331·0)	-0·1 (-2·8–3·2)
Russia	4 890 000 (3 990 000–6 070 000)	7.8 (4.1–12.3)	2 710 (2 180–3 400)	-0·2 (-0·4–-0·1)	481 000 (326 000–688 000)	269·0 (182·0–380·0)	0·0 (-0·6–0·7)
Ukraine	1 510 000 (1 240 000–1 890 000)	-7.3 (-9.9–-4.3)	2 710 (2 180–3 410)	-0·1 (-0·2–-0·1)	149 000 (102 000–214 000)	270·0 (182·0–382·0)	-0·1 (-2·9–2·2)
HIGH INCOME	35 200 000 (28 000 000–43 100 000)	28.0 (22.9–32.8)	2 560 (2 060–3 170)	-3·8 (-5·1–-2·7)	3 470 000 (2 360 000–4 730 000)	256·0 (170·0–357·0)	-4·0 (-5·5–-2·7)
**Australasia**	494 000 (399 000–612 000)	66.5 (59.5–73.8)	1 360 (1 070–1 670)	0·0 (-0·1–0·1)	48 800 (33 100–66 700)	135·0 (90·4–188·0)	-0·0 (-3·6–2·6)
Australia	440 000 (356 000–542 000)	66.6 (59.7–74.0)	1 450 (1 130–1 770)	0·0 (-0·1–0·2)	43 300 (29 400–59 500)	144·0 (96·6–201·0)	-0·1 (-4·2–3·0)
New Zealand	54 900 (42 800–69 900)	65.3 (56.8–73.6)	915 (693–1 160)	0·1 (-0·2–0·5)	5 420 (3 550–7 940)	91·2 (58·5–130·0)	0·6 (-2·6–4·6)
**High income Asia Pacific**	5 750 000 (4 650 000–7 100 000)	26.4 (18.3–36.4)	2 260 (1 810–2 870)	-2·3 (-3·3–-1·6)	573 000 (387 000–802 000)	229·0 (153·0–328·0)	-2·1 (-3·2–-1·0)

Brunei	9 540<br>(7 500–12 100)	125.0<br>(108.0–139.0)	2 010<br>(1 610–2 520)	0·3<br>(0·1–0·4)	970<br>(630–1 390)	203·0<br>(134·0–290·0)	0·4<br>(-2·7–3·7)
Japan	4 240 000<br>(3 450 000–5 240 000)	16.0<br>(8.8–25.8)	2 450<br>(1 950–3 110)	0·0<br>(-0·1–0·2)	421 000<br>(287 000–587 000)	249·0<br>(166·0–356·0)	0·3<br>(-0·4–1·2)
South Korea	1 350 000<br>(1 080 000–1 700 000)	64.3<br>(46.5–82.1)	1 890<br>(1 530–2 370)	-0·9<br>(-1·1–-0·5)	135 000<br>(89 500–195 000)	190·0<br>(128·0–273·0)	-0·9<br>(-4·1–2·3)
Singapore	149 000<br>(118 000–186 000)	130.0<br>(110.0–149.0)	2 010<br>(1 610–2 530)	-0·4<br>(-0·6–-0·1)	15 000<br>(9 960–21 800)	204·0<br>(136·0–294·0)	-0·1<br>(-2·3–3·2)
**High income North America**	10 300 000<br>(8 310 000–12 700 000)	40.1<br>(33.0–46.9)	2 380<br>(1 880–3 000)	-0·2<br>(-0·3–-0·1)	1 010 000<br>(679 000–1 410 000)	235·0<br>(158·0–336·0)	-1·1<br>(-1·7–-0·2)
Canada	965 000<br>(784 000–1 170 000)	48.4<br>(38.5–57.5)	2 150<br>(1 740–2 640)	-0·0<br>(-0·1–0·0)	95 700<br>(64 200–132 000)	215·0<br>(143·0–307·0)	-0·2<br>(-2·9–3·3)
Greenland	1 380<br>(1 100–1 730)	15.6<br>(4.9–26.6)	2 130<br>(1 730–2 630)	0·7<br>(0·2–1·1)	137<br>(91–196)	212·0<br>(140·0–303·0)	0·8<br>(-2·3–3·8)
USA	9 370 000<br>(7 520 000–11 600 000)	40.6<br>(33.8–47.4)	2 400<br>(1 910–3 030)	-0·2<br>(-0·3–-0·1)	916 000<br>(619 000–1 290 000)	237·0<br>(159·0–340·0)	-1·1<br>(-1·9–-0·4)
SOUTHERN LATIN AMERICA	1 570 000<br>(1 250 000–1 990 000)	55.9<br>(51.4–59.4)	2 100<br>(1 680–2 670)	-0·0<br>(-0·1–-0·0)	157 000<br>(105 000–218 000)	210·0<br>(139·0–295·0)	-0·4<br>(-2·5–1·7)
Argentina	1 040 000<br>(828 000–1 320 000)	53.4<br>(50.7–55.4)	2 110<br>(1 680–2 670)	-0·0<br>(-0·0–0·0)	104 000<br>(69 600–145 000)	211·0<br>(140·0–296·0)	-0·3<br>(-3·0–2·6)
Chile	448 000<br>(355 000–566 000)	71.5<br>(60.9–81.0)	2 100<br>(1 670–2 670)	-0·2<br>(-0·2–-0·1)	44 500<br>(29 600–61 600)	210·0<br>(137·0–294·0)	-0·5<br>(-3·6–2·4)
Uruguay	83 900<br>(66 700–106 000)	20.7<br>(18.1–23.2)	2 110<br>(1 680–2 680)	0·1<br>(0·0–0·1)	8 320<br>(5 570–11 400)	211·0<br>(139·0–296·0)	-0·2<br>(-3·4–2·9)
WESTERN EUROPE	17 100 000<br>(13 800 000–21 200 000)	19.5<br>(14.9–24.0)	2 970<br>(2 370–3 670)	-4·5<br>(-6·6–-2·5)	1 680 000<br>(1 140 000–2 270 000)	297·0<br>(196·0–413·0)	-4·4<br>(-6·7–-2·4)
Andorra	3 490<br>(2 780–4 430)	91.5<br>(74.8–105.0)	2 990<br>(2 350–3 740)	0·5<br>(0·3–0·6)	347<br>(226–489)	299·0<br>(196·0–423·0)	0·1<br>(-2·3–2·6)
Austria	446 000<br>(353 000–556 000)	27.5<br>(22.9–31.3)	3 760<br>(2 980–4 640)	-0·5<br>(-0·8–-0·2)	44 100<br>(28 900–60 700)	376·0<br>(247·0–528·0)	-0·4<br>(-2·4–1·3)

Belgium	449 000 (352 000–544 000)	23.1 (19.4–26.8)	3 040 (2 410–3 740)	-0·1 (-0·2–0·0)	44 100 (29 200–59 800)	303·0 (201·0–422·0)	-0·3 (-2·4–1·9)
Cyprus	50 400 (39 700–63 300)	106.0 (97.5–112.0)	3 020 (2 370–3 780)	0·3 (0·1–0·5)	5 020 (3 270–7 170)	303·0 (199·0–432·0)	0·4 (-1·9–2·9)
Denmark	274 000 (216 000–341 000)	20.1 (15.3–23.9)	3 660 (2 870–4 600)	-0·1 (-0·3–-0·0)	27 100 (17 500–35 500)	366·0 (236·0–500·0)	-0·0 (-2·2–1·8)
Finland	184 000 (145 000–227 000)	23.1 (12.7–34.8)	2 530 (1 970–3 140)	1·9 (-5·7–9·3)	18 100 (12 000–23 700)	252·0 (167·0–351·0)	1·8 (-6·0–9·3)
France	1 960 000 (1 590 000–2 460 000)	27.6 (22.1–33.1)	2 310 (1 850–2 910)	-0·0 (-0·1–0·1)	192 000 (130 000–255 000)	231·0 (154·0–318·0)	0·0 (-2·5–3·5)
Germany	3 470 000 (2 760 000–4 360 000)	15.9 (11.7–20.5)	3 000 (2 360–3 760)	-0·6 (-0·8–-0·3)	341 000 (225 000–464 000)	299·0 (196·0–424·0)	-0·6 (-3·2–2·4)
Greece	509 000 (398 000–621 000)	13.7 (9.1–18.7)	3 630 (2 860–4 530)	0·0 (-0·0–0·0)	50 200 (32 900–67 000)	363·0 (238·0–497·0)	-0·2 (-2·1–2·0)
Iceland	12 600 (10 000–15 900)	59.8 (52.4–66.6)	3 000 (2 350–3 760)	-0·2 (-0·3–-0·1)	1 260 (817–1 740)	301·0 (195·0–428·0)	0·0 (-1·9–2·0)
Ireland	180 000 (142 000–225 000)	62.8 (55.0–68.0)	3 010 (2 370–3 770)	0·1 (0·0–0·2)	17 800 (11 600–24 800)	300·0 (197·0–427·0)	-0·2 (-2·3–2·0)
Israel	298 000 (234 000–372 000)	110.0 (103.0–115.0)	3 010 (2 360–3 770)	-0·4 (-0·5–-0·2)	29 800 (19 500–41 700)	302·0 (198·0–433·0)	-0·2 (-2·5–1·9)
Italy	2 730 000 (2 200 000–3 380 000)	19.8 (13.2–26.0)	3 280 (2 670–4 020)	-0·3 (-0·4–-0·2)	269 000 (181 000–372 000)	329·0 (224·0–458·0)	0·1 (-0·7–0·9)
Luxembourg	24 200 (19 300–30 600)	74.9 (70.6–78.3)	3 000 (2 350–3 750)	-0·5 (-0·7–-0·2)	2 410 (1 580–3 400)	301·0 (196·0–426·0)	-0·4 (-2·9–1·9)
Malta	17 700 (14 100–22 200)	42.7 (34.9–53.9)	3 000 (2 360–3 760)	-0·7 (-1·0–-0·4)	1 750 (1 150–2 370)	301·0 (194·0–428·0)	-0·8 (-3·1–1·7)
Monaco	1 600 (1 260–2 020)	25.8 (21.4–29.1)	3 020 (2 370–3 780)	-0·2 (-0·4–-0·0)	158 (104–212)	301·0 (196·0–426·0)	-0·7 (-3·3–1·5)
Netherlands	599 000 (474 000–752 000)	29.5 (22.4–35.8)	2 670 (2 150–3 370)	-0·1 (-0·3–0·1)	59 200 (38 500–79 400)	267·0 (179·0–366·0)	-0·3 (-3·3–2·6)

Norway	138 000 (110 000–177 000)	18.1 (10.9–26.0)	2 060 (1 670–2 640)	-11·7 (-17·2–-7·0)	13 700 (9 400–19 700)	206·0 (139·0–301·0)	-11·2 (-16·8–-6·6)
Portugal	444 000 (354 000–556 000)	26.0 (19.7–32.3)	3 030 (2 370–3 790)	-0·1 (-0·2–-0·0)	43 600 (29 000–59 200)	302·0 (200·0–430·0)	0·1 (-2·3–2·9)
San Marino	1 310 (1 040–1 630)	59.4 (53.4–65.0)	3 030 (2 380–3 790)	0·8 (0·4–1·2)	129 (84–178)	303·0 (198·0–433·0)	0·3 (-2·0–3·0)
Spain	1 530 000 (1 210 000–1 930 000)	-6.8 (-21.1–8.9)	2 460 (1 970–3 080)	-32·3 (-43·0–-20·5)	151 000 (99 000–212 000)	246·0 (164·0–345·0)	-32·3 (-42·3–-21·2)
Sweden	421 000 (340 000–514 000)	24.1 (21.5–26.8)	3 210 (2 550–3 980)	-0·2 (-0·8–0·6)	41 700 (27 700–56 900)	322·0 (211·0–446·0)	-0·0 (-2·2–2·3)
Switzerland	351 000 (281 000–441 000)	40.2 (35.9–44.5)	3 000 (2 360–3 760)	-0·3 (-0·5–-0·1)	34 600 (22 700–47 800)	300·0 (197·0–427·0)	-0·2 (-2·7–2·4)
UK	2 960 000 (2 420 000–3 660 000)	21.1 (15.1–28.4)	3 500 (2 810–4 260)	-1·1 (-5·8–5·7)	292 000 (199 000–396 000)	349·0 (232·0–481·0)	-1·1 (-5·3–5·6)
LATIN AMERICA AND CARIBBEAN	16 800 000 (13 400 000–21 100 000)	93.9 (82.1–105.0)	2 690 (2 150–3 350)	0·1 (-0·0–0·2)	1 670 000 (1 140 000–2 400 000)	267·0 (182·0–380·0)	-0·0 (-0·6–0·5)
**Andean Latin America**	1 590 000 (1 250 000–2 010 000)	112.0 (101.0–122.0)	2 480 (1 960–3 110)	-0·0 (-0·1–0·1)	159 000 (106 000–228 000)	248·0 (166·0–353·0)	-0·3 (-2·4–1·3)
Bolivia	278 000 (216 000–350 000)	131.0 (123.0–138.0)	2 470 (1 960–3 110)	-0·3 (-0·4–-0·2)	27 800 (18 400–39 900)	247·0 (166·0–351·0)	-0·6 (-3·0–1·9)
Ecuador	435 000 (342 000–549 000)	121.0 (110.0–132.0)	2 480 (1 960–3 120)	0·1 (-0·0–0·2)	43 800 (29 100–63 000)	249·0 (167·0–356·0)	0·0 (-2·8–2·8)
Peru	874 000 (688 000–1 110 000)	103.0 (90.8–114.0)	2 480 (1 960–3 120)	0·0 (-0·1–0·2)	87 700 (57 900–126 000)	248·0 (165·0–353·0)	-0·4 (-3·7–2·1)
**Caribbean**	1 260 000 (998 000–1 580 000)	60.0 (52.5–67.4)	2 480 (1 970–3 120)	0·0 (-0·0–0·1)	125 000 (85 200–179 000)	247·0 (168·0–354·0)	-0·3 (-1·4–1·0)
Antigua and Barbuda	2 570 (2 040–3 280)	83.3 (71.1–94.6)	2 480 (1 970–3 120)	-0·3 (-0·4–-0·1)	256 (171–369)	248·0 (166·0–353·0)	-0·7 (-3·0–2·4)
Bahamas	10 600 (8 400–13 600)	86.1 (72.1–100.0)	2 480 (1 970–3 120)	-0·1 (-0·1–-0·0)	1 060 (700–1 520)	248·0 (165·0–351·0)	-0·6 (-3·2–1·7)

Barbados	9 220<br>(7 380–11 700)	41.3<br>(30.3–51.3)	2 480<br>(1 970–3 120)	-0·2<br>(-0·4–-0·1)	914<br>(619–1 300)	249·0<br>(168·0–354·0)	-0·9<br>(-3·9–1·8)
Belize	9 800<br>(7 600–12 400)	195.0<br>(180.0–207.0)	2 470<br>(1 960–3 110)	0·3<br>(0·2–0·6)	987<br>(650–1 440)	248·0<br>(168·0–357·0)	-0·3<br>(-2·8–2·3)
Bermuda	2 100<br>(1 660–2 640)	26.0<br>(14.3–37.2)	2 480<br>(1 970–3 110)	-0·2<br>(-0·4–-0·1)	208<br>(140–296)	249·0<br>(169·0–354·0)	-0·4<br>(-2·8–2·1)
Cuba	355 000<br>(283 000–451 000)	28.3<br>(18.3–39.4)	2 470<br>(1 960–3 110)	0·0<br>(-0·1–0·2)	35 300<br>(23 800–50 300)	248·0<br>(168·0–357·0)	-0·0<br>(-2·6–2·4)
Dominica	1 920<br>(1 520–2 420)	16.5<br>(11.0–22.3)	2 460<br>(1 950–3 090)	-0·8<br>(-1·4–-0·2)	191<br>(128–269)	246·0<br>(165·0–353·0)	-1·3<br>(-3·9–2·1)
Dominican Republic	270 000<br>(211 000–341 000)	90.2<br>(79.2–99.8)	2 470<br>(1 960–3 110)	-0·2<br>(-0·4–-0·1)	27 100<br>(18 000–38 900)	247·0<br>(165·0–353·0)	-0·8<br>(-3·3–2·2)
Grenada	2 820<br>(2 230–3 600)	59.1<br>(48.9–68.8)	2 470<br>(1 950–3 100)	-0·7<br>(-1·1–-0·4)	282<br>(190–406)	246·0<br>(166·0–356·0)	-1·0<br>(-3·6–1·8)
Guyana	19 200<br>(14 900–24 500)	24.8<br>(17.3–31.7)	2 480<br>(1 970–3 120)	0·1<br>(0·0–0·2)	1 910<br>(1 270–2 710)	245·0<br>(166·0–347·0)	-0·5<br>(-3·3–2·8)
Haiti	275 000<br>(212 000–347 000)	130.0<br>(125.0–134.0)	2 490<br>(1 970–3 130)	0·2<br>(0·0–0·5)	27 500<br>(17 900–40 100)	246·0<br>(168·0–352·0)	0·3<br>(-2·3–2·5)
Jamaica	75 900<br>(59 600–95 800)	52.4<br>(44.9–58.7)	2 480<br>(1 960–3 110)	-0·2<br>(-0·3–-0·1)	7 620<br>(5 130–10 900)	248·0<br>(168·0–356·0)	-0·5<br>(-3·1–2·4)
Puerto Rico	110 000<br>(87 500–138 000)	22.3<br>(14.1–31.4)	2 490<br>(1 970–3 130)	-0·0<br>(-0·1–0·0)	10 900<br>(7 510–15 200)	249·0<br>(168·0–353·0)	-0·3<br>(-2·8–2·4)
Saint Kitts and Nevis	1 730<br>(1 370–2 210)	92.3<br>(74.1–108.0)	2 470<br>(1 960–3 100)	-0·5<br>(-0·8–-0·2)	172<br>(115–252)	246·0<br>(166·0–354·0)	-0·8<br>(-3·8–1·4)
Saint Lucia	5 150<br>(4 100–6 540)	87.4<br>(70.5–102.0)	2 470<br>(1 960–3 110)	-0·5<br>(-0·7–-0·3)	512<br>(343–742)	247·0<br>(166·0–352·0)	-0·7<br>(-3·6–1·8)
Saint Vincent and the Grenadines	3 140<br>(2 490–3 960)	42.9<br>(30.0–55.0)	2 460<br>(1 950–3 090)	-0·6<br>(-1·0–-0·2)	312<br>(210–443)	246·0<br>(165·0–350·0)	-1·3<br>(-3·7–1·5)
Suriname	15 300<br>(12 100–19 400)	82.8<br>(71.3–94.3)	2 480<br>(1 970–3 120)	0·2<br>(0·1–0·4)	1 520<br>(1 020–2 160)	246·0<br>(166·0–350·0)	-0·5<br>(-3·3–2·6)

Trinidad and Tobago	40 500<br>(32 600–51 400)	52.6<br>(42.0–64.0)	2 470<br>(1 960–3 110)	-0·1<br>(-0·2–-0·1)	4 020<br>(2 750–5 790)	246·0<br>(167·0–354·0)	-0·3<br>(-3·1–2·2)
Virgin Islands	3 130<br>(2 480–3 950)	21.5<br>(10.8–33.6)	2 490<br>(1 980–3 130)	0·1<br>(-0·0–0·2)	309<br>(210–433)	249·0<br>(168·0–351·0)	-0·4<br>(-3·8–2·2)
CENTRAL LATIN AMERICA	6 980 000<br>(5 570 000–8 780 000)	101.0<br>(87.4–113.0)	2 680<br>(2 150–3 350)	-0·0<br>(-0·2–0·1)	697 000<br>(470 000–1 000 000)	267·0<br>(181·0–381·0)	-0·0<br>(-0·8–0·8)
Colombia	1 300 000<br>(1 030 000–1 650 000)	93.5<br>(81.0–107.0)	2 480<br>(1 970–3 120)	0·2<br>(-0·0–0·5)	131 000<br>(87 300–184 000)	249·0<br>(167·0–352·0)	0·4<br>(-2·4–2·9)
Costa Rica	130 000<br>(103 000–165 000)	109.0<br>(95.2–122.0)	2 480<br>(1 970–3 130)	0·4<br>(0·2–0·6)	13 000<br>(8 760–18 400)	248·0<br>(167·0–350·0)	-0·1<br>(-2·8–3·1)
El Salvador	157 000<br>(124 000–199 000)	54.8<br>(46.5–60.9)	2 500<br>(1 990–3 160)	0·7<br>(0·3–1·1)	15 700<br>(10 500–22 200)	250·0<br>(168·0–353·0)	0·8<br>(-1·8–3·7)
Guatemala	401 000<br>(309 000–504 000)	147.0<br>(140.0–151.0)	2 490<br>(1 980–3 140)	0·5<br>(0·2–0·8)	40 000<br>(26 200–57 100)	247·0<br>(168·0–350·0)	0·3<br>(-2·4–2·7)
Honduras	219 000<br>(170 000–275 000)	173.0<br>(165.0–179.0)	2 490<br>(1 970–3 130)	0·4<br>(0·2–0·6)	22 000<br>(14 300–31 300)	248·0<br>(166·0–351·0)	-0·0<br>(-2·4–3·1)
Mexico	3 760 000<br>(3 040 000–4 720 000)	95.8<br>(80.9–109.0)	2 870<br>(2 330–3 570)	0·1<br>(0·0–0·2)	376 000<br>(255 000–543 000)	286·0<br>(194·0–410·0)	0·1<br>(-0·5–0·8)
Nicaragua	154 000<br>(119 000–195 000)	136.0<br>(122.0–146.0)	2 480<br>(1 970–3 130)	0·0<br>(-0·2–0·3)	15 400<br>(10 200–22 300)	248·0<br>(168·0–353·0)	-0·2<br>(-3·2–2·7)
Panama	107 000<br>(84 600–135 000)	112.0<br>(101.0–123.0)	2 470<br>(1 960–3 110)	0·1<br>(0·0–0·3)	10 700<br>(7 130–15 200)	247·0<br>(165·0–352·0)	-0·5<br>(-3·2–1·6)
Venezuela	743 000<br>(592 000–940 000)	96.4<br>(79.7–113.0)	2 480<br>(1 970–3 120)	0·1<br>(0·1–0·2)	74 300<br>(50 300–107 000)	248·0<br>(169·0–353·0)	0·1<br>(-2·4–2·5)
TROPICAL LATIN AMERICA	6 990 000<br>(5 540 000–8 740 000)	90.7<br>(78.8–103.0)	2 790<br>(2 220–3 480)	0·1<br>(-0·0–0·2)	692 000<br>(470 000–989 000)	277·0<br>(188·0–392·0)	0·0<br>(-0·7–0·9)
Brazil	6 820 000<br>(5 410 000–8 530 000)	90.1<br>(78.1–102.0)	2 800<br>(2 230–3 490)	0·1<br>(0·0–0·2)	675 000<br>(459 000–965 000)	278·0<br>(188·0–394·0)	0·1<br>(-0·7–0·9)
Paraguay	168 000<br>(133 000–211 000)	120.0<br>(114.0–126.0)	2 450<br>(1 980–3 050)	-0·1<br>(-0·1–-0·0)	16 800<br>(11 300–24 300)	244·0<br>(166·0–346·0)	-0·4<br>(-2·3–1·7)

NORTH AFRICA AND MIDDLE EAST	22 200 000 (17 500 000–28 400 000)	143.0 (132.0–151.0)	3 750 (3 010–4 720)	-0·8 (-1·1–-0·6)	2 210 000 (1 470 000–3 210 000)	370·0 (251·0–533·0)	-1·4 (-2·1–-0·7)
Afghanistan	905 000 (706 000–1 170 000)	197.0 (178.0–218.0)	3 710 (2 950–4 660)	-1·9 (-3·0–-0·7)	89 300 (59 600–129 000)	360·0 (243·0–521·0)	-3·1 (-5·5–-0·4)
Algeria	1 580 000 (1 240 000–2 010 000)	151.0 (133.0–162.0)	3 700 (2 940–4 660)	-0·7 (-0·9–-0·3)	157 000 (103 000–229 000)	366·0 (244·0–524·0)	-0·9 (-2·7–1·5)
Bahrain	60 300 (46 500–79 700)	348.0 (298.0–389.0)	3 260 (2 630–4 060)	-3·5 (-4·5–-2·4)	6 000 (3 900–8 780)	321·0 (219·0–462·0)	-4·1 (-6·5–-2·3)
Egypt	3 220 000 (2 510 000–4 080 000)	112.0 (108.0–115.0)	3 630 (2 880–4 570)	-1·8 (-2·1–-1·4)	320 000 (211 000–463 000)	359·0 (238·0–519·0)	-2·2 (-4·0–-0·0)
Iran	4 100 000 (3 290 000–5 240 000)	142.0 (123.0–154.0)	4 410 (3 520–5 540)	0·7 (0·4–1·1)	405 000 (274 000–606 000)	434·0 (293·0–633·0)	0·4 (-0·4–1·1)
Iraq	1 320 000 (1 030 000–1 670 000)	221.0 (213.0–230.0)	3 690 (2 930–4 640)	-0·1 (-0·1–0·0)	130 000 (86 900–189 000)	360·0 (242·0–518·0)	-1·1 (-3·4–1·4)
Jordan	381 000 (298 000–486 000)	356.0 (328.0–374.0)	3 570 (2 840–4 480)	-1·9 (-2·3–-1·5)	37 900 (24 900–55 500)	352·0 (235·0–507·0)	-2·5 (-4·1–-0·3)
Kuwait	178 000 (136 000–233 000)	312.0 (279.0–336.0)	3 310 (2 650–4 170)	8·9 (7·0–10·5)	17 700 (11 500–26 400)	327·0 (220·0–471·0)	7·9 (4·8–10·9)
Lebanon	209 000 (165 000–264 000)	109.0 (101.0–117.0)	3 820 (3 030–4 820)	1·9 (1·2–2·5)	20 500 (13 600–29 800)	374·0 (251·0–541·0)	1·3 (-1·1–3·8)
Libya	271 000 (211 000–350 000)	179.0 (157.0–196.0)	3 660 (2 910–4 600)	3·4 (2·7–4·0)	26 800 (17 400–39 100)	360·0 (241·0–519·0)	2·4 (-0·2–4·7)
Morocco	1 390 000 (1 090 000–1 770 000)	99.8 (89.0–108.0)	3 720 (2 950–4 680)	-0·5 (-0·6–-0·3)	137 000 (90 700–202 000)	365·0 (243·0–531·0)	-1·4 (-3·7–1·0)
Oman	141 000 (108 000–182 000)	215.0 (199.0–225.0)	3 250 (2 610–4 040)	-0·3 (-0·9–0·2)	14 200 (9 010–20 900)	321·0 (216·0–461·0)	-0·6 (-2·6–1·8)
Palestine	142 000 (111 000–180 000)	208.0 (199.0–217.0)	3 700 (2 940–4 660)	-3·1 (-3·9–-2·4)	14 100 (9 360–20 400)	364·0 (244·0–526·0)	-3·7 (-6·4–-1·4)
Qatar	90 800 (69 800–119 000)	703.0 (675.0–725.0)	2 870 (2 330–3 580)	-4·4 (-5·5–-3·4)	9 130 (5 720–13 800)	283·0 (188·0–406·0)	-5·1 (-7·4–-3·1)

Saudi Arabia	1 320 000 (1 010 000–1 730 000)	265.0 (237.0–283.0)	3 370 (2 700–4 230)	0·3 (-0·2–0·7)	132 000 (84 500–196 000)	332·0 (222·0–478·0)	-0·0 (-2·1–2·1)
Sudan	1 130 000 (882 000–1 440 000)	132.0 (128.0–136.0)	3 680 (2 920–4 640)	-0·5 (-0·8–-0·2)	113 000 (74 400–163 000)	363·0 (242·0–525·0)	-0·8 (-2·7–1·3)
Syria	548 000 (433 000–710 000)	90.0 (74.5–101.0)	3 780 (3 000–4 770)	3·1 (2·5–4·0)	54 100 (35 900–78 700)	373·0 (250·0–543·0)	2·6 (0·5–5·0)
Tunisia	500 000 (395 000–636 000)	111.0 (97.4–120.0)	3 740 (2 970–4 710)	1·1 (0·9–1·4)	49 300 (32 600–72 200)	369·0 (245·0–535·0)	0·3 (-1·8–2·7)
Türkiye	3 540 000 (2 790 000–4 500 000)	102.0 (91.9–110.0)	3 710 (2 950–4 680)	-0·1 (-0·2–-0·0)	351 000 (230 000–512 000)	368·0 (243·0–531·0)	-0·4 (-2·4–2·0)
United Arab Emirates	356 000 (265 000–473 000)	698.0 (601.0–773.0)	2 920 (2 360–3 630)	-4·2 (-5·5–-2·8)	35 800 (22 400–55 400)	289·0 (196·0–415·0)	-4·1 (-7·1–-1·6)
Yemen	845 000 (661 000–1 070 000)	195.0 (189.0–201.0)	3 720 (2 960–4 680)	0·2 (-0·5–0·7)	83 600 (55 200–120 000)	364·0 (245·0–526·0)	0·3 (-2·1–2·7)
SOUTH ASIA	27 600 000 (22 200 000–34 600 000)	112.0 (105.0–118.0)	1 590 (1 290–1 970)	0·4 (0·2–0·5)	2 740 000 (1 880 000–3 980 000)	157·0 (109·0–228·0)	0·9 (0·1–1·7)
Bangladesh	2 150 000 (1 720 000–2 690 000)	120.0 (106.0–132.0)	1 380 (1 110–1 700)	1·3 (0·9–1·7)	214 000 (148 000–312 000)	136·0 (93·9–197·0)	1·5 (-1·9–5·1)
Bhutan	9 900 (7 860–12 400)	76.7 (65.6–85.0)	1 370 (1 100–1 690)	-0·2 (-0·4–-0·0)	992 (649–1 470)	136·0 (90·2–196·0)	-0·0 (-3·0–3·8)
India	22 300 000 (17 900 000–27 900 000)	76.7 (65.6–85.0)	1 620 (1 310–2 000)	0·3 (0·1–0·4)	2 210 000 (1 510 000–3 210 000)	160·0 (111·0–232·0)	1·0 (0·1–1·7)
Nepal	384 000 (305 000–481 000)	105.0 (98.7–110.0)	1 390 (1 120–1 720)	1·3 (0·9–1·6)	38 100 (25 500–55 000)	137·0 (91·3–197·0)	1·8 (-1·2–5·7)
Pakistan	2 790 000 (2 200 000–3 550 000)	126.0 (122.0–130.0)	1 610 (1 310–1 990)	0·6 (0·4–0·7)	278 000 (188 000–401 000)	159·0 (111·0–230·0)	0·1 (-2·1–2·7)
SOUTHEAST ASIA, EAST ASIA, AND OCEANIA	67 300 000 (54 400 000–83 600 000)	85.8 (69.1–102.0)	2 520 (2 010–3 130)	2·1 (-2·5–5·8)	6 730 000 (4 500 000–9 560 000)	253·0 (170·0–357·0)	2·2 (-2·3–6·1)

**East Asia**	49 900 000 (40 000 000–62 000 000)	80.5 (61.7–100.0)	2 560 (2 040–3 170)	2·6 (-3·4–7·6)	4 980 000 (3 340 000–7 120 000)	257·0 (173·0–362·0)	2·7 (-3·0–7·9)
China	48 000 000 (38 400 000–60 000 000)	80.8 (61.5–101.0)	2 550 (2 030–3 180)	2·9 (-3·2–8·0)	4 800 000 (3 210 000–6 880 000)	256·0 (172·0–362·0)	2·9 (-2·9–8·5)
North Korea	826 000 (650 000–1 040 000)	66.3 (60.5–72.5)	2 550 (2 020–3 170)	-1·3 (-1·8–-0·9)	82 800 (54 600–122 000)	256·0 (170·0–375·0)	-1·4 (-4·0–0·9)
Taiwan (province of China)	977 000 (917 000–1 050 000)	78.9 (44.6–118.0)	2 810 (2 640–3 030)	-1·4 (-20·4–17·4)	97 100 (71 500–124 000)	281·0 (208·0–358·0)	-1·7 (-21·1–18·6)
**Oceania**	259 000 (209 000–328 000)	138.0 (132.0–143.0)	2 430 (1 990–3 010)	0·1 (-0·0–0·1)	26 000 (17 500–37 300)	240·0 (164·0–335·0)	0·2 (-1·7–2·2)
American Samoa	1 300 (1 060–1 640)	55.2 (44.7–65.7)	2 440 (2 000–3 030)	0·9 (0·6–1·2)	129 (85.8–177)	241·0 (164·0–331·0)	-0·1 (-2·8–2·2)
Cook Islands	530 (431–660)	41.1 (29.9–51.5)	2 450 (2 010–3 040)	1·3 (0·9–1·9)	52.2 (34.8–71.5)	243·0 (164·0–338·0)	0·6 (-2·1–3·1)
Fiji	21 800 (17 700–27 200)	57.9 (49.0–67.7)	2 440 (2 000–3 020)	0·1 (-0·0–0·3)	2 170 (1 480–3 080)	242·0 (165·0–337·0)	-0·1 (-2·6–2·2)
Guam	4 480 (3 650–5 630)	61.6 (48.6–73.9)	2 430 (1 990–3 010)	0·5 (0·3–0·8)	448 (299–622)	243·0 (167·0–340·0)	0·2 (-2·4–2·4)
Kiribati	2 410 (1 930–3 020)	84.9 (79.8–88.5)	2 470 (2 030–3 060)	0·4 (0·3–0·6)	241 (163–344)	244·0 (169·0–341·0)	0·8 (-1·3–3·5)
Marshall Islands	1 210 (972–1 530)	90.2 (78.2–100.0)	2 430 (1 990–3 010)	0·1 (-0·2–0·4)	121 (81.4–174)	240·0 (164·0–337·0)	-0·5 (-2·8–2·1)
Micronesia (Federated States of)	2 240 (1 810–2 810)	39.0 (30.8–45.9)	2 440 (2 000–3 030)	0·5 (0·3–0·6)	224 (152–315)	242·0 (166·0–335·0)	0·4 (-2·1–3·4)
Nauru	191 (153–243)	17.3 (15.7–19.0)	2 460 (2 020–3 050)	1·9 (1·3–2·6)	19.2 (12.7–27.8)	243·0 (166·0–339·0)	1·0 (-1·6–3·9)
Niue	47.6 (38.7–60)	-6.2 (-10.1–-2.7)	2 440 (2 000–3 030)	-0·3 (-0·6–-0·2)	4.7 (3.13–6.45)	242·0 (166·0–336·0)	-0·8 (-3·1–1·7)
Northern Mariana Islands	1 350 (1 060–1 760)	45.0 (21.6–73.0)	2 420 (1 980–3 010)	2·5 (1·5–3·4)	134 (86.5–190)	242·0 (166·0–340·0)	2·1 (-0·8–5·1)

Palau	561 (450–723)	76.8 (57.9–91.8)	2 410 (1 970–2 990)	-1·0 (-1·6–-0·6)	55.4 (36.5–78.9)	238·0 (162·0–331·0)	-1·6 (-4·1–1·3)
Papua New Guinea	187 000 (150 000–237 000)	175.0 (168.0–180.0)	2 420 (1 980–3 000)	-0·1 (-0·2–0·1)	18 800 (12 600–27 100)	240·0 (163·0–334·0)	0·4 (-2·1–3·0)
Samoa	4 340 (3 510–5 440)	57.3 (51.8–61.5)	2 430 (1 990–3 010)	-0·2 (-0·4–0·0)	433 (292–610)	241·0 (163·0–336·0)	-0·5 (-3·3–2·7)
Solomon Islands	11 900 (9 620–15 200)	138.0 (128.0–145.0)	2 440 (1 990–3 020)	1·1 (0·7–1·5)	1 200 (808–1 730)	242·0 (165·0–336·0)	0·8 (-1·6–3·5)
Tokelau	33.8 (27.7–42.5)	3.1 (-1.7–7.0)	2 430 (1 990–3 020)	-1·7 (-2·3–-1·1)	3.35 (2.27–4.69)	241·0 (166·0–339·0)	-2·1 (-4·5–1·0)
Tonga	2 140 (1 750–2 680)	27.0 (22.4–30.4)	2 450 (2 010–3 040)	-0·0 (-0·2–0·2)	214 (147–295)	244·0 (167·0–337·0)	-0·4 (-3·0–2·1)
Tuvalu	277 (226–344)	40.4 (37.5–42.8)	2 440 (2 000–3 020)	-1·9 (-2·7–-1·3)	27.6 (18.5–38.5)	242·0 (164·0–337·0)	-2·2 (-4·4–-0·0)
Vanuatu	5 660 (4 540–7 070)	138.0 (132.0–144.0)	2 430 (1 990–3 000)	0·7 (0·4–0·9)	567 (385–798)	241·0 (165·0–337·0)	0·3 (-2·0–2·9)
SOUTHEAST ASIA	17 200 000 (13 600 000–21 900 000)	102.0 (91.0–112.0)	2 400 (1 920–3 010)	0·1 (-0·2–0·4)	1 720 000 (1 140 000–2 490 000)	240·0 (161·0–341·0)	0·5 (-0·3–1·1)
Cambodia	343 000 (276 000–432 000)	133.0 (125.0–141.0)	2 250 (1 810–2 820)	-0·7 (-1·1–-0·3)	34 400 (23 000–48 600)	223·0 (150·0–313·0)	0·1 (-2·5–2·7)
Indonesia	6 950 000 (5 430 000–8 940 000)	92.1 (81.4–100.0)	2 550 (2 030–3 210)	-0·2 (-0·3–-0·1)	700 000 (456 000–1 040 000)	256·0 (169·0–369·0)	0·1 (-0·9–1·0)
Laos	138 000 (111 000–175 000)	122.0 (116.0–126.0)	2 220 (1 790–2 790)	-0·5 (-0·7–-0·3)	14 000 (9 470–20 200)	222·0 (151·0–314·0)	0·2 (-2·2–2·4)
Malaysia	708 000 (571 000–889 000)	158.0 (139.0–182.0)	2 210 (1 780–2 770)	6·4 (-0·6–16·1)	70 800 (47 200–101 000)	220·0 (147·0–310·0)	6·5 (-0·6–15·7)
Maldives	11 200 (8 990–14 200)	279.0 (248.0–312.0)	2 150 (1 730–2 700)	-1·6 (-2·5–-0·6)	1 140 (735–1 640)	215·0 (143·0–300·0)	-1·2 (-4·1–1·8)
Mauritius	36 600 (29 700–46 400)	71.5 (55.9–86.2)	2 220 (1 790–2 790)	-0·2 (-0·3–-0·1)	3 600 (2 450–5 010)	220·0 (152·0–306·0)	-0·4 (-3·6–3·6)

Myanmar	1 230 000 (987 000–1 570 000)	80.9 (71.7–88.8)	2 250 (1 810–2 820)	0·8 (0·6–1·0)	123 000 (83 000–173 000)	224·0 (152·0–310·0)	1·2 (-2·4–4·5)
Philippines	2 590 000 (2 030 000–3 320 000)	128.0 (119.0–136.0)	2 560 (2 030–3 220)	-0·0 (-0·1–0·2)	260 000 (171 000–382 000)	255·0 (169·0–370·0)	0·2 (-0·4–0·8)
Seychelles	2 650 (2 130–3 370)	94.1 (80.1–106.0)	2 200 (1 770–2 760)	-1·2 (-1·5–-0·9)	265 (176–374)	219·0 (148·0–306·0)	-1·7 (-4·1–1·0)
Sri Lanka	563 000 (457 000–705 000)	75.9 (63.2–88.5)	2 240 (1 800–2 810)	1·0 (0·7–1·4)	55 600 (37 100–77 400)	221·0 (148·0–308·0)	0·7 (-2·0–3·2)
Thailand	2 120 000 (1 740 000–2 680 000)	95.2 (76.5–113.0)	2 230 (1 790–2 800)	0·1 (-0·0–0·1)	211 000 (141 000–300 000)	223·0 (151·0–315·0)	0·5 (-2·0–2·8)
Timor-Leste	22 600 (18 200–28 500)	101.0 (90.3–113.0)	2 210 (1 780–2 780)	0·2 (0·1–0·4)	2 270 (1 560–3 200)	220·0 (151·0–311·0)	0·4 (-3·0–2·8)
Viet Nam	2 430 000 (1 940 000–3 060 000)	118.0 (102.0–130.0)	2 230 (1 800–2 800)	-0·8 (-1·1–-0·6)	244 000 (162 000–345 000)	224·0 (151·0–312·0)	-0·3 (-4·0–3·1)
SUB-SAHARAN AFRICA	21 500 000 (17 200 000–27 600 000)	145.0 (140.0–149.0)	2 750 (2 220–3 490)	1·4 (0·1–2·6)	2 160 000 (1 430 000–3 150 000)	272·0 (183·0–395·0)	1·7 (0·3–2·8)
**Central sub-Saharan Africa**	2 450 000 (1 950 000–3 140 000)	160.0 (157.0–163.0)	2 580 (2 090–3 320)	-0·5 (-0·7–-0·2)	244 000 (164 000–354 000)	254·0 (172·0–369·0)	0·1 (-1·9–2·1)
Angola	543 000 (432 000–695 000)	211.0 (207.0–214.0)	2 610 (2 110–3 360)	1·9 (1·4–2·3)	54 300 (36 300–78 300)	258·0 (175·0–375·0)	2·0 (-0·2–4·5)
Central African Republic	101 000 (79 900–129 000)	107.0 (105.0–110.0)	2 590 (2 090–3 330)	-0·4 (-0·7–-0·1)	9 980 (6 620–14 300)	254·0 (170·0–366·0)	-0·2 (-2·2–2·3)
Congo	114 000 (90 600–148 000)	170.0 (156.0–182.0)	2 570 (2 080–3 300)	-1·3 (-1·8–-0·8)	11 400 (7 600–16 500)	254·0 (172·0–366·0)	-1·4 (-4·0–1·4)
Democratic Republic of the Congo	1 630 000 (1 300 000–2 090 000)	150.0 (147.0–154.0)	2 570 (2 080–3 310)	-1·2 (-1·6–-0·7)	162 000 (109 000–236 000)	253·0 (172·0–368·0)	-0·4 (-3·3–2·2)
Equatorial Guinea	26 200 (20 700–33 500)	251.0 (235.0–268.0)	2 590 (2 100–3 350)	-1·6 (-2·4–-1·1)	2 630 (1 720–3 850)	255·0 (172·0–373·0)	-1·0 (-4·2–1·3)
Gabon	40 200 (32 000–52 300)	118.0 (111.0–125.0)	2 590 (2 100–3 330)	0·7 (0·2–1·2)	4 000 (2 690–5 790)	256·0 (173·0–369·0)	0·5 (-2·0–3·9)

**Eastern sub-Saharan Africa**	6 330 000 (5 020 000–8 020 000)	143.0 (140.0–146.0)	2 220 (1 790–2 790)	0·1 (0·0–0·2)	636 000 (422 000–943 000)	220·0 (150·0–325·0)	0·7 (-0·2–1·5)
Burundi	167 000 (132 000–211 000)	129.0 (125.0–131.0)	2 080 (1 690–2 610)	-1·4 (-2·0–-0·7)	16 800 (11 000–24 700)	206·0 (139·0–302·0)	-1·3 (-3·8–2·2)
Comoros	13 300 (10 700–16 900)	110.0 (101.0–116.0)	2 090 (1 700–2 640)	-0·4 (-0·7–-0·2)	1 330 (901–2 020)	208·0 (142·0–313·0)	-0·3 (-3·4–2·8)
Djibouti	21 800 (17 300–28 000)	257.0 (235.0–273.0)	2 070 (1 690–2 600)	0·1 (-0·2–0·5)	2 200 (1 430–3 280)	207·0 (141·0–308·0)	0·3 (-2·4–3·0)
Eritrea	103 000 (81 100–132 000)	167.0 (163.0–170.0)	2 090 (1 710–2 640)	-0·6 (-0·8–-0·3)	10 400 (6 660–15 500)	207·0 (140·0–307·0)	0·1 (-3·0–3·1)
Ethiopia	1 770 000 (1 400 000–2 250 000)	131.0 (127.0–135.0)	2 400 (1 950–3 000)	-0·1 (-0·2–0·1)	178 000 (120 000–264 000)	238·0 (163·0–352·0)	0·6 (-0·8–1·9)
Kenya	926 000 (735 000–1 180 000)	181.0 (173.0–188.0)	2 400 (1 950–3 010)	-0·0 (-0·2–0·1)	93 200 (62 600–138 000)	239·0 (163·0–353·0)	0·0 (-0·7–0·7)
Madagascar	407 000 (321 000–518 000)	156.0 (151.0–161.0)	2 090 (1 710–2 640)	-0·0 (-0·2–0·1)	41 100 (27 400–61 300)	208·0 (139·0–310·0)	0·7 (-2·2–3·7)
Malawi	264 000 (208 000–330 000)	110.0 (107.0–113.0)	2 090 (1 710–2 650)	-0·0 (-0·1–0·1)	26 500 (17 400–38 900)	208·0 (140·0–304·0)	0·7 (-2·1–3·7)
Mozambique	398 000 (314 000–500 000)	116.0 (110.0–122.0)	2 100 (1 720–2 660)	-0·0 (-0·2–0·2)	39 500 (25 900–58 700)	206·0 (139·0–308·0)	-0·1 (-3·2–3·3)
Rwanda	206 000 (163 000–261 000)	121.0 (116.0–125.0)	2 100 (1 720–2 670)	-0·1 (-0·2–0·0)	20 700 (13 500–30 900)	209·0 (141·0–313·0)	0·1 (-2·8–2·7)
Somalia	271 000 (213 000–341 000)	186.0 (178.0–195.0)	2 090 (1 710–2 650)	-0·1 (-0·3–0·3)	27 200 (17 600–40 300)	207·0 (139·0–304·0)	0·2 (-3·1–3·3)
South Sudan	132 000 (105 000–166 000)	71.6 (64.5–78.4)	2 100 (1 710–2 640)	1·9 (1·1–2·5)	13 100 (8 640–19 700)	206·0 (140·0–311·0)	1·9 (-1·5–5·0)
Uganda	543 000 (429 000–681 000)	156.0 (152.0–159.0)	2 100 (1 720–2 660)	0·5 (0·2–0·8)	54 400 (35 600–80 200)	208·0 (142·0–312·0)	1·4 (-0·8–4·4)
Tanzania	838 000 (662 000–1 060 000)	148.0 (142.0–152.0)	2 100 (1 710–2 650)	-0·0 (-0·0–0·0)	84 200 (56 000–125 000)	208·0 (142·0–306·0)	1·1 (-1·5–3·7)

Zambia	264 000 (208 000–334 000)	167.0 (160.0–174.0)	2 080 (1 700–2 630)	-0·5 (-0·8–-0·1)	26 500 (17 200–39 700)	206·0 (138·0–306·0)	-0·4 (-3·5–2·7)
**Southern sub-Saharan Africa**	2 150 000 (1 720 000–2 740 000)	88.2 (80.8–93.7)	2 880 (2 320–3 640)	-0·0 (-0·3–0·2)	212 000 (142 000–314 000)	283·0 (192·0–417·0)	-0·9 (-1·7–0·1)
Botswana	57 200 (45 100–74 600)	148.0 (134.0–158.0)	2 590 (2 090–3 340)	-0·8 (-1·1–-0·6)	5 680 (3 810–8 080)	255·0 (173·0–366·0)	-1·7 (-4·1–1·4)
Eswatini	24 100 (19 100–31 000)	82.2 (76.5–86.8)	2 610 (2 110–3 360)	-0·3 (-0·8–0·1)	2 380 (1 590–3 380)	255·0 (175·0–367·0)	-2·3 (-4·6–0·9)
Lesotho	47 500 (37 600–61 400)	40.0 (36.6–43.7)	2 590 (2 090–3 340)	0·0 (-0·4–0·4)	4 670 (3 150–6 680)	252·0 (172·0–363·0)	-1·9 (-4·0–1·1)
Namibia	53 200 (42 400–68 700)	108.0 (100.0–113.0)	2 610 (2 110–3 350)	0·5 (0·3–0·7)	5 300 (3 520–7 600)	257·0 (174·0–372·0)	-0·1 (-2·7–2·7)
South Africa	1 670 000 (1 330 000–2 120 000)	90.2 (82.4–96.4)	2 970 (2 400–3 740)	-0·4 (-0·5–-0·3)	164 000 (111 000–245 000)	291·0 (198·0–429·0)	-1·3 (-2·1–-0·3)
Zimbabwe	301 000 (238 000–385 000)	76.8 (70.7–81.3)	2 620 (2 110–3 370)	1·2 (0·6–1·8)	30 000 (19 900–43 700)	258·0 (174·0–372·0)	0·4 (-2·5–3·9)
**Western sub-Saharan Africa**	10 600 000 (8 370 000–13 600 000)	159.0 (152.0–166.0)	3 240 (2 610–4 100)	3·0 (0·4–5·6)	1 070 000 (700 000–1 530 000)	321·0 (214·0–458·0)	3·4 (0·5–5·5)
Benin	262 000 (204 000–338 000)	188.0 (184.0–192.0)	3 030 (2 440–3 880)	-1·1 (-1·4–-0·8)	26 300 (17 100–37 000)	300·0 (202·0–418·0)	-0·5 (-3·2–1·8)
Burkina Faso	480 000 (375 000–619 000)	156.0 (151.0–161.0)	3 070 (2 470–3 930)	-0·4 (-0·7–-0·1)	48 200 (32 000–67 900)	305·0 (203·0–423·0)	0·4 (-2·1–3·4)
Cabo Verde	16 400 (13 100–20 900)	120.0 (107.0–130.0)	2 980 (2 400–3 820)	-5·7 (-6·8–-4·4)	1 640 (1 080–2 310)	297·0 (198·0–411·0)	-6·2 (-8·5–-3·8)
Cameroon	646 000 (503 000–834 000)	212.0 (207.0–218.0)	3 000 (2 420–3 840)	-0·6 (-0·7–-0·4)	64 700 (43 000–91 100)	297·0 (199·0–408·0)	-0·3 (-2·5–2·3)
Chad	301 000 (234 000–383 000)	157.0 (152.0–163.0)	2 980 (2 410–3 820)	-2·5 (-3·0–-2·0)	30 100 (19 700–42 500)	295·0 (198·0–413·0)	-2·7 (-4·8–-0·3)
Côte d’Ivoire	582 000 (454 000–754 000)	155.0 (147.0–161.0)	2 930 (2 360–3 750)	0·1 (-0·2–0·3)	58 300 (38 400–82 000)	290·0 (193·0–398·0)	1·2 (-1·0–3·8)

The Gambia	49 100 (38 200–63 100)	168.0 (165.0–172.0)	3 000 (2 420–3 840)	2·4 (1·9–3·0)	4 900 (3 200–6 840)	297·0 (199·0–409·0)	1·9 (-0·5–4·2)
Ghana	801 000 (628 000–1 030 000)	163.0 (156.0–167.0)	3 060 (2 460–3 920)	1·8 (1·4–2·1)	80 500 (52 400–114 000)	305·0 (203·0–422·0)	2·2 (-1·2–5·2)
Guinea	266 000 (208 000–341 000)	104.0 (100.0–109.0)	3 040 (2 450–3 900)	0·4 (0·1–0·5)	26 600 (17 500–36 800)	302·0 (202·0–415·0)	0·5 (-2·0–3·0)
Guinea-Bissau	41 500 (32 300–53 500)	111.0 (108.0–115.0)	3 060 (2 460–3 910)	-0·3 (-0·5–0·0)	4 160 (2 670–5 810)	302·0 (201·0–416·0)	-0·2 (-2·4–2·0)
Liberia	112 000 (87 400–145 000)	172.0 (161.0–181.0)	2 960 (2 380–3 790)	-0·5 (-0·8–-0·2)	11 100 (7 290–15 500)	288·0 (195·0–395·0)	-0·4 (-3·1–2·4)
Mali	433 000 (337 000–555 000)	148.0 (143.0–154.0)	2 990 (2 410–3 830)	-0·9 (-1·1–-0·7)	43 300 (28 000–60 100)	296·0 (197·0–409·0)	-0·8 (-3·4–1·1)
Mauritania	92 900 (73 000–120 000)	121.0 (118.0–123.0)	3 020 (2 430–3 870)	0·1 (-0·1–0·3)	9 360 (6 100–13 000)	302·0 (203·0–420·0)	0·5 (-1·9–3·1)
Niger	419 000 (325 000–534 000)	189.0 (184.0–195.0)	3 030 (2 440–3 880)	1·9 (1·3–2·5)	42 200 (27 100–59 100)	302·0 (199·0–420·0)	2·2 (0·3–5·0)
Nigeria	5 400 000 (4 250 000–6 890 000)	158.0 (146.0–171.0)	3 490 (2 790–4 370)	6·4 (1·3–11·6)	541 000 (358 000–793 000)	346·0 (230·0–502·0)	6·7 (1·6–11·7)
São Tomé and Príncipe	5 110 (4 020–6 650)	113.0 (104.0–122.0)	2 980 (2 400–3 820)	-1·7 (-2·0–-1·3)	514 (334–728)	297·0 (198·0–412·0)	-1·8 (-4·2–0·4)
Senegal	345 000 (271 000–445 000)	136.0 (133.0–138.0)	3 020 (2 440–3 870)	0·2 (-0·1–0·4)	34 600 (22 800–47 900)	300·0 (201·0–413·0)	0·3 (-2·1–2·5)
Sierra Leone	184 000 (143 000–237 000)	137.0 (132.0–141.0)	2 980 (2 400–3 810)	-0·8 (-1·0–-0·6)	18 400 (12 100–25 800)	294·0 (197·0–408·0)	-0·7 (-3·7–1·8)
Togo	191 000 (150 000–248 000)	179.0 (168.0–188.0)	3 060 (2 460–3 910)	-0·1 (-0·5–0·3)	19 200 (12 500–27 100)	304·0 (203·0–421·0)	0·1 (-2·2–2·1)

Supplemental Table 6. Forecast of neck pain age-standardised prevalence (%) and total cases (in millions) globally and by region, both sexes, to 2050, with 95% uncertainty intervals

	**Age-standardised prevalence (%)**			**Cases (millions)**		
**Region**	**2030**	**2040**	**2050**	**2030**	**2040**	**2050**
Global	2·38 (1·91–2·97)	2·30 (1·83–2·90)	2·22 (1·74–2·81)	233 (187–284)	256 (207–309)	269 (219–322)
Andean Latin America	2·48 (1·96–3·11)	2·47 (1·96–3·11)	2·47 (1·96–3·11)	1·88 (1·50–2·33)	2·14 (1·72–2·61)	2·36 (1·87–2·86)
Australasia	1·36 (1·07–1·67)	1·36 (1·07–1·67)	1·36 (1·07–1·67)	0·555 (0·453–0·689)	0·617 (0·499–0·776)	0·671 (0·538–0·860)
Caribbean	2·48 (1·96–3·12)	2·48 (1·96–3·12)	2·48 (1·96–3·11)	1·37 (1·08–1·72)	1·45 (1·14–1·80)	1·49 (1·17–1·83)
Central Asia	2·33 (1·86–2·91)	2·33 (1·85–2·91)	2·33 (1·85–2·90)	2·53 (1·96–3·17)	2·81 (2·21–3·49)	3·05 (2·39–3·76)
Central Europe	2·45 (1·96–3·08)	2·45 (1·96–3·08)	2·45 (1·96–3·08)	3·53 (2·85–4·35)	3·37 (2·75–4·13)	3·15 (2·59–3·86)
Central Latin America	2·68 (2·15–3·35)	2·67 (2·14–3·35)	2·67 (2·14–3·34)	8·05 (6·47–9·94)	8·88 (7·14–10·8)	9·39 (7·51–11·2)
Central sub-Saharan Africa	2·58 (2·09–3·32)	2·58 (2·09–3·32)	2·58 (2·09–3·32)	3·24 (2·61–4·10)	4·16 (3·40–5·16)	5·15 (4·24–6·26)
East Asia	2·56 (2·04–3·17)	2·55 (2·03–3·16)	2·54 (2·03–3·15)	55·8 (44·5–68·1)	57·1 (46·5–68·1)	53·8 (43·6–64·9)
Eastern Europe	2·67 (2·15–3·36)	2·67 (2·15–3·36)	2·67 (2·15–3·36)	6·90 (5·59–8·56)	6·71 (5·45–8·29)	6·51 (5·35–8·06)
Eastern sub-Saharan Africa	2·22 (1·80–2·79)	2·22 (1·80–2·79)	2·21 (1·79–2·79)	8·65 (6·81–10·8)	11·5 (9·02–14·0)	14·6 (11·4–17·5)
High income Asia Pacific	2·26 (1·81–2·86)	2·25 (1·80–2·85)	2·25 (1·80–2·85)	5·75 (4·71–7·03)	5·53 (4·57–6·69)	5·13 (4·22–6·24)
High income North America	2·37 (1·88–2·99)	2·35 (1·88–2·97)	2·34 (1·86–2·96)	11·3 (8·92–14·0)	12·1 (9·10–15·0)	12·5 (9·54–15·5)
North Africa and Middle East	3·74 (3·00–4·71)	3·73 (2·98–4·69)	3·71 (2·96–4·68)	27·9 (22·4–34·7)	33·1 (26·5–40·6)	37·3 (29·9–45·4)
Oceania	2·43 (1·99–3·01)	2·44 (1·99–3·01)	2·43 (1·99–3·01)	0·341 (0·274–0·422)	0·437 (0·352–0·536)	0·549 (0·441–0·678)

South Asia	1·59 (1·29–1·97)	1·59 (1·29–1·97)	1·59 (1·29–1·97)	34·0 (27·2–41·1)	40·3 (32·8–47·3)	45·2 (37·4–52·5)
Southeast Asia	2·41 (1·94–3·02)	2·43 (1·96–3·04)	2·45 (1·97–3·06)	21·7 (17·6–26·4)	25·5 (20·6–30·4)	28·0 (22·9–33·0)
Southern Latin America	2·10 (1·67–2·67)	2·10 (1·67–2·67)	2·10 (1·67–2·66)	1·78 (1·43–2·21)	1·95 (1·58–2·39)	2·05 (1·67–2·51)
Southern sub-Saharan Africa	2·87 (2·32–3·63)	2·87 (2·31–3·62)	2·86 (2·30–3·62)	2·51 (2·00–3·16)	2·86 (2·28–3·56)	3·18 (2·53–3·91)
Tropical Latin America	2·79 (2·21–3·47)	2·79 (2·21–3·47)	2·78 (2·21–3·47)	7·97 (6·46–9·75)	8·62 (6·99–10·4)	8·90 (7·17–10·6)
Western Europe	2·98 (2·38–3·68)	2·98 (2·37–3·67)	2·98 (2·35–3·67)	17·7 (14·5–22·0)	18·0 (14·7–22·5)	18·0 (14·5–22·4)
Western sub-Saharan Africa	3·24 (2·61–4·10)	3·24 (2·61–4·11)	3·24 (2·61–4·10)	14·2 (11·2–18·0)	18·6 (14·6–23·3)	23·6 (18·4–29·1)

Supplemental Figure 3: Forecast to 2050 of neck pain age-standardised prevalence for GBD super-regions

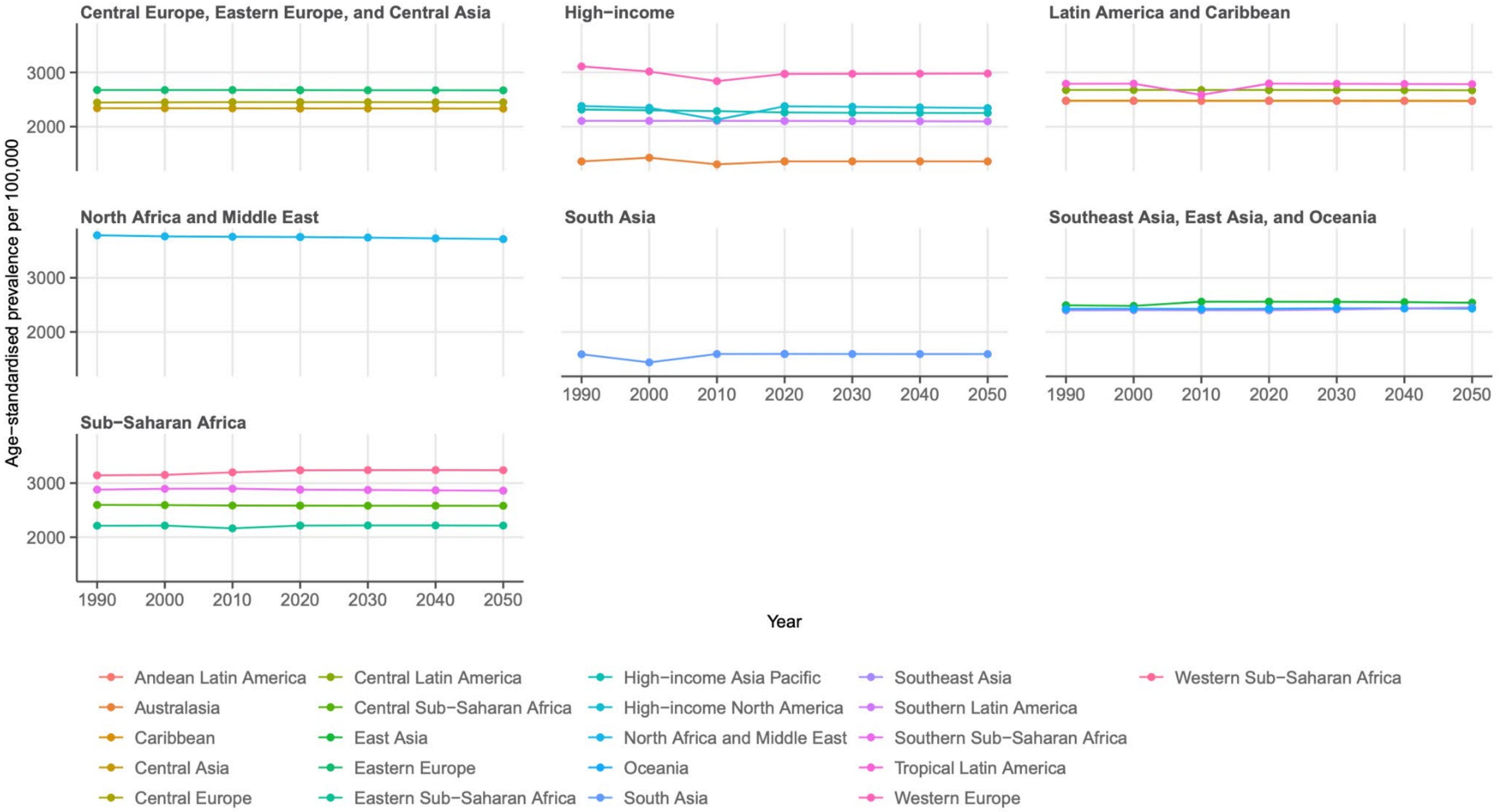

Neck Pain GBD 2021 Data input sources

National Center for Health Statistics (NCHS), Centers for Disease Control and Prevention (CDC), US Census Bureau. United States National Health Interview Survey 2002. Hyattsville, United States: National Center for Health Statistics (NCHS), Centers for Disease Control and Prevention (CDC).

National Center for Health Statistics (NCHS), Centers for Disease Control and Prevention (CDC), US Census Bureau. United States National Health Interview Survey 2003. Hyattsville, United States: National Center for Health Statistics (NCHS), Centers for Disease Control and Prevention (CDC).

National Center for Health Statistics (NCHS), Centers for Disease Control and Prevention (CDC), US Census Bureau. United States National Health Interview Survey 2005. Hyattsville, United States: National Center for Health Statistics (NCHS), Centers for Disease Control and Prevention (CDC).

National Center for Health Statistics (NCHS), Centers for Disease Control and Prevention (CDC), US Census Bureau. United States National Health Interview Survey 2006. Hyattsville, United States: National Center for Health Statistics (NCHS), Centers for Disease Control and Prevention (CDC).

National Center for Health Statistics (NCHS), Centers for Disease Control and Prevention (CDC), US Census Bureau. United States National Health Interview Survey 2007. Hyattsville, United States: National Center for Health Statistics (NCHS), Centers for Disease Control and Prevention (CDC).

National Center for Health Statistics (NCHS), Centers for Disease Control and Prevention (CDC), US Census Bureau. United States National Health Interview Survey 2008. Hyattsville, United States: National Center for Health Statistics (NCHS), Centers for Disease Control and Prevention (CDC).

National Center for Health Statistics (NCHS), Centers for Disease Control and Prevention (CDC), US Census Bureau. United States National Health Interview Survey 2009. Hyattsville, United States: National Center for Health Statistics (NCHS), Centers for Disease Control and Prevention (CDC).

National Center for Health Statistics (NCHS), Centers for Disease Control and Prevention (CDC). United States National Health and Nutrition Examination Survey 2003-2004. Hyattsville, United States: National Center for Health Statistics (NCHS), Centers for Disease Control and Prevention (CDC).

National Center for Health Statistics (NCHS), Centers for Disease Control and Prevention (CDC). United States National Health and Nutrition Examination Survey 2009-2010. Hyattsville, United States: National Center for Health Statistics (NCHS), Centers for Disease Control and Prevention (CDC), 2011.

National Center for Health Statistics (NCHS), Centers for Disease Control and Prevention (CDC). United States National Health and Nutrition Examination Survey 2001-2002. Hyattsville, United States: National Center for Health Statistics (NCHS), Centers for Disease Control and Prevention (CDC).

National Center for Health Statistics (NCHS), Centers for Disease Control and Prevention (CDC). United States National Health and Nutrition Examination Survey 1999-2000. Hyattsville, United States: National Center for Health Statistics (NCHS), Centers for Disease Control and Prevention (CDC).

NatCen Social Research and University College London. Department of Epidemiology and Public Health, Health Survey for England, 2011 [computer file]. Colchester, Essex: UK Data Archive [distributor], April 2013. SN: 7260, http://dx.doi.org/10.5255/UKDA-SN-7260-1

Mäkelä M, Heliövaara M, Sievers K, Impivaara O, Knekt P, Aromaa A. Prevalence, determinants, and consequences of chronic neck pain in Finland. Am J Epidemiol. 1991; 134(11): 1356-67.

Bassols A, Bosch F, Campillo M, Cañellas M, Baños JE. An epidemiological comparison of pain complaints in the general population of Catalonia (Spain). Pain. 1999; 83(1): 9-16.

Bergman S, Herrström P, Högström K, Petersson IF, Svensson B, Jacobsson LT. Chronic musculoskeletal pain, prevalence rates, and sociodemographic associations in a Swedish population study. J Rheumatol. 2001; 28(6): 1369-77.

Bovim G, Schrader H, Sand T. Neck pain in the general population. Spine. 1994; 19(12): 1307-9.

Côté P, Cassidy JD, Carroll L. The Saskatchewan Health and Back Pain Survey. The prevalence of neck pain and related disability in Saskatchewan adults. Spine. 1998; 23(15): 1689-98.

Diepenmaat ACM, van der Wal MF, de Vet HCW, Hirasing RA. Neck/shoulder, low back, and arm pain in relation to computer use, physical activity, stress, and depression among Dutch adolescents. Pediatrics. 2006; 117(2): 412-6.

Hakala PT, Rimpelä AH, Saarni LA, Salminen JJ. Frequent computer-related activities increase the risk of neck-shoulder and low back pain in adolescents. Eur J Public Health. 2006; 16(5): 536-41.

Hartvigsen J, Frederiksen H, Christensen K. Back and neck pain in seniors-prevalence and impact. Eur Spine J. 2006; 15(6): 802-6.

Davatchi F, Jamshidi A-R, Banihashemi AT, Gholami J, Forouzanfar MH, Akhlaghi M, Barghamdi M, Noorolahzadeh E, Khabazi A-R, Salesi M, Salari A-H, Karimifar M, Essalat-Manesh K, Hajialiloo M, Soroosh M, Farzad F, Moussavi H-R, Samadi F, Ghaznavi K, Asgharifard H, Zangiabadi A-H, Shahram F, Nadji A, Akbarian M, Gharibdoost F. WHO-ILAR COPCORD Study (Stage 1, Urban Study) in Iran. J Rheumatol. 2008; 35(7): 1384.

Lacey RJ, Lewis M, Sim J. Presentation of pain drawings in questionnaire surveys: influence on prevalence of neck and upper limb pain in the community. Pain. 2003; 105(1-2): 293-301.

Lau EM, Sham A, Wong KC. The prevalence of and risk factors for neck pain in Hong Kong Chinese. J Public Health Med. 1996; 18(4): 396-9.

Fejer R, Jordan A, Hartvigsen J. Categorising the severity of neck pain: establishment of cut-points for use in clinical and epidemiological research. Pain. 2005; 119(1-3): 176-82.

Ståhl M, Kautiainen H, El-Metwally A, Häkkinen A, Ylinen J, Salminen JJ, Mikkelsson M. Non-specific neck pain in schoolchildren: prognosis and risk factors for occurrence and persistence. A 4-year follow-up study. Pain. 2008; 137(2): 316-22.

Palm P, Hansson Risberg E, Mortimer M, Palmerud G, Toomingas A, Wigaeus Tornqvist E. Computer use, neck and upper-extremity symptoms, eyestrain and headache among female and male upper secondary school students. Scand J Work Environ Health Suppl. 2007; 3(3): 33-41.

Palmer KT, Walker-Bone K, Griffin MJ, Syddall H, Pannett B, Coggon D, Cooper C. Prevalence and occupational associations of neck pain in the British population. Scand J Work Environ Health. 2001; 27(1): 49-56.

Cardon G, De Bourdeaudhuij I, De Clercq D, Philippaerts RM, Verstraete S, Geldhof E. Physical Fitness, Physical Activity, and Self-Reported Back and Neck Pain in Elementary Schoolchildren. Pediatr Exerc Sci. 2004; 16(2): 147-157.

Picavet HSJ, Schouten JSAG. Musculoskeletal pain in the Netherlands: prevalences, consequences and risk groups, the DMC(3)-study. Pain. 2003; 102(1-2): 167-78.

Sim J, Lacey RJ, Lewis M. The impact of workplace risk factors on the occurrence of neck and upper limb pain: a general population study. BMC Public Health. 2006; 6(1): 234.

Siivola SM, Levoska S, Latvala K, Hoskio E, Vanharanta H, Keinänen-Kiukaanniemi S. Predictive factors for neck and shoulder pain: a longitudinal study in young adults. Spine. 2004; 29(15): 1662-9.

Auvinen J, Tammelin T, Taimela S, Zitting P, Karppinen J. Neck and shoulder pains in relation to physical activity and sedentary activities in adolescence. Spine. 2007; 32(9): 1038-44.

Thomas E, Peat G, Harris L, Wilkie R, Croft PR. The prevalence of pain and pain interference in a general population of older adults: cross-sectional findings from the North Staffordshire Osteoarthritis Project (NorStOP). Pain. 2004; 110(1-2): 361-8.

Urwin M, Symmons D, Allison T, Brammah T, Busby H, Roxby M, Simmons A, Williams G. Estimating the burden of musculoskeletal disorders in the community: the comparative prevalence of symptoms at different anatomical sites, and the relation to social deprivation. Ann Rheum Dis. 1998; 57(11): 649-55.

Vikat A, Rimpelä M, Salminen JJ, Rimpelä A, Savolainen A, Virtanen SM. Neck or shoulder pain and low back pain in Finnish adolescents. Scand J Public Health. 2000; 28(3): 164-73.

Von Korff M, Crane P, Lane M, Miglioretti DL, Simon G, Saunders K, Stang P, Brandenburg N, Kessler R. Chronic spinal pain and physical-mental comorbidity in the United States: results from the national comorbidity survey replication. Pain. 2005; 113(3): 331-9.

Webb R, Brammah T, Lunt M, Urwin M, Allison T, Symmons D. Prevalence and predictors of intense, chronic, and disabling neck and back pain in the UK general population. Spine. 2003; 28(11): 1195-202.

Westerling D, Jonsson BG. Pain from the neck-shoulder region and sick leave. Scand J Soc Med. 1980; 8(3): 131-6.

Friedrich M, Rustler T, Hahne J. Prevalence of self-reported musculoskeletal pain in the Austrian population. Wien Klin Wochenschr. 2006; 118(3-4): 82-9.

Al-Awadhi AM, Olusi SO, Moussa M, Shehab D, Al-Zaid N, Al-Herz A, Al-Jarallah K. Musculoskeletal pain, disability and health-seeking behavior in adult Kuwaitis using a validated Arabic version of the WHO-ILAR COPCORD Core Questionnaire. Clin Exp Rheumatol. 2004; 22(2): 177-83.

Joshi VL, Chopra A. Is there an urban-rural divide? Population surveys of rheumatic musculoskeletal disorders in the Pune region of India using the COPCORD Bhigwan model. J Rheumatol. 2009; 36(3): 614-22.

Veerapen K, Wigley RD, Valkenburg H. Musculoskeletal pain in Malaysia: a COPCORD survey. J Rheumatol. 2007; 34(1): 207-13.

Ministry of Health (New Zealand), National Research Bureau Ltd (New Zealand). New Zealand Health Survey 2006-2007.

Dolphens M, Cagnie B, Coorevits P, Vanderstraeten G, Cardon G, D'hooge R, Danneels L. Sagittal standing posture and its association with spinal pain: a school-based epidemiological study of 1196 Flemish adolescents before age at peak height velocity. Spine. 2012; 37(19): 1657-66.

Kjaer P, Wedderkopp N, Korsholm L, Leboeuf-Yde C. Prevalence and tracking of back pain from childhood to adolescence. BMC Musculoskelet Disord. 2011; 98.

Stranjalis G, Kalamatianos T, Stavrinou LC, Tsamandouraki K, Alamanos Y. Neck pain in a sample of Greek urban population (fifteen to sixty-five years): analysis according to personal and socioeconomic characteristics. Spine. 2011; 36(16): E1098-1104.

Statistics Norway. Norway Survey of Living Conditions 2008-2009. Oslo, Norway: Statistics Norway.

Statistics Norway. Norway Survey of Living Conditions 2005-2006. Oslo, Norway: Statistics Norway.

Statistics Norway. Norway Survey of Living Conditions 2002. Oslo, Norway: Statistics Norway.

Statistics Norway. Norway Survey of Living Conditions 2012-2013.

Stahl MK, El-Metwally AA, Rimpela AH. Time trends in single versus concomitant neck and back pain in Finnish adolescents: results from national cross-sectional surveys from 1991 to 2011. BMC Musculoskelet Disord. 2014; 296.

Aartun E, Hartvigsen J, Wedderkopp N, Hestbaek L. Spinal pain in adolescents: prevalence, incidence, and course: a school-based two-year prospective cohort study in 1,300 Danes aged 11-13. BMC Musculoskelet Disord. 2014; 187.

Mesas AE, Gonzalez AD, Mesas CE, de Andrade SM, Magro IS, del Llano J. The association of chronic neck pain, low back pain, and migraine with absenteeism due to health problems in Spanish workers. Spine. 2014; 39(15): 1243-53.

Shan Z, Deng G, Li J, Li Y, Zhang Y, Zhao Q. Correlational analysis of neck/shoulder pain and low back pain with the use of digital products, physical activity and psychological status among adolescents in Shanghai. PLoS One. 2013; 8(10): e78109.

Woo J, Leung J, Lau E. Prevalence and correlates of musculoskeletal pain in Chinese elderly and the impact on 4-year physical function and quality of life. Public Health. 2009; 123(8): 549-56.

Hartvigsen J, Christensen K. Pain in the back and neck are with us until the end: a nationwide interview-based survey of Danish 100-year-olds. Spine. 2008; 33(8): 909-13.

Jimenez-Sanchez S, Fernandez-de-Las-Penas C, Carrasco-Garrido P, Hernandez-Barrera V, Alonso-Blanco C, Palacios-Cena D, Jimenez-Garcia R. Prevalence of chronic head, neck and low back pain and associated factors in women residing in the Autonomous Region of Madrid (Spain). Gac Sanit. 2012; 26(6): 534-40.

Gao HL, Lin SQ, Wei Y, Chen Y, Wu ZL. The effect of age and menopausal status on musculoskeletal symptoms in Chinese women aged 35-64 years. Climacteric. 2013; 16(6): 639-45.

El-Sayed AM, Hadley C, Tessema F, Tegegn A, Cowan JA, Galea S. Back and neck pain and psychopathology in rural sub-Saharan Africa: evidence from the Gilgel Gibe Growth and Development Study, Ethiopia. Spine. 2010; 35(6): 684-9.

Skillgate E, Magnusson C, Lundberg M, Hallqvist J. The age- and sex-specific occurrence of bothersome neck pain in the general population--results from the Stockholm public health cohort. BMC Musculoskelet Disord. 2012; 185.

Son KM, Cho NH, Lim SH, Kim HA. Prevalence and risk factor of neck pain in elderly Korean community residents. J Korean Med Sci. 2013; 28(5): 680–6.

Goode AP, Freburger J, Carey T. Prevalence, practice patterns, and evidence for chronic neck pain. Arthritis Care Res (Hoboken). 2010; 62(11): 1594–601.

Leclerc A, Chastang JF, Taiba R, Pascal P, Cyr D, Plouvier S, Descatha A. Musculoskeletal pain at various anatomical sites and socioeconomic position: Results of a national survey. Rev Epidemiol Sante Publique. 2016; 64(5): nan.

Dolphens M, Vansteelandt S, Cagnie B, Vleeming A, Nijs J, Vanderstraeten G, Danneels L. Multivariable modeling of factors associated with spinal pain in young adolescence. Eur Spine J. 2016; 25(9.0): 2809-21.

Noormohammadpour P, Mansournia MA, Koohpayehzadeh J, Asgari F, Rostami M, Rafei A, Kordi R. Prevalence of chronic neck pain, low back pain and knee pain and their related factors in community-dwelling adults in Iran: A population-based national study. Clin J Pain. 2016; 33(2): 181-7.

Ogwumike OO, Adeniyi AF, Orogbemi OO. Musculoskeletal pain among postmenopausal women in Nigeria: Association with overall and central obesity. Hong Kong Physiother J. 2016; 34: 41–6.

Statistics Norway. Norway Long-term Illness: Disease, Injury or Impairment by Sex and Age. Oslo, Norway: Statistics Norway.

Statistics Norway. Norway Survey of Living Conditions on Health 2015.

Depintor JD, Bracher ES, Cabral DM, Eluf-Neto J. Prevalence of chronic spinal pain and identification of associated factors in a sample of the population of Sao Paulo, Brazil: cross-sectional study. Sao Paulo Med J. 2016; 134(5): 375-384.

Bikbov MM, Fayzrakhmanov RR, Kazakbaeva GM, Zainullin RM, Salavatova VF, Gilmanshin TR, Arslangareeva II, Nikitin NA, Mukhamadieva SR, Yakupova DF, Khikmatullin RI, Aminev SK, Nuriev IF, Zaynetdinov AF, Uzianbaeva YV, Jonas JB. Prevalence and Associated Factors of Low Backpain, Thoracic Spine Pain and Neck Pain in Russians: The Ural Eye and Medical Study. [Unpublished].

Genebra CVDS, Maciel NM, Bento TPF, Simeão SFAP, Vitta AD. Prevalence and Factors Associated with Neck Pain: a Population-based Study. Braz J Phys Ther. 2017; 21(4): 274–80.

D.G. Hoy, T. Raikoti, E. Smith, A. Tuzakana, T. Gill, K. Matikarai, J. Tako, A. Jorari, F. Blyth, A. Pitaboe, R. Buchbinder, I. Kalauma, P. Brooks, C. Lepers, A. Woolf, A. Briggs, and L. March. Use of The Global Alliance for Musculoskeletal Health survey module for estimating the population prevalence of musculoskeletal pain: findings from the Solomon Islands [Unpublished]. BMC Musculoskelet Disord. 2018; 19(292).

Alok R, Srivastava R, Kumar P, Das SK, Agarwal GG, Dhaon P. Prevalence of rheumatic musculoskeletal symptoms in rural and urban areas: a cross-sectional study in northern India. Int J Rheum Dis. 2017; 20(11): 1638-1647.

Ben Ayed H, Yaich S, Trigui M, Ben Hmida M, Ben Jemaa M, Ammar A, Jedidi J, Karray R, Feki H, Mejdoub Y, Kassis M, Damak J. Prevalence, Risk Factors and Outcomes of Neck, Shoulders and Low-Back Pain in Secondary-School Children. J Res Health Sci. 2019; 19(1): e00440.

Dianat I, Alipour A, Asgari Jafarabadi M. Risk factors for neck and shoulder pain among schoolchildren and adolescents. J Paediatr Child Health. 2018; 54(1): 20-27.

Gonçalves TR, Mediano MFF, Sichieri R, Cunha DB. Is Health-related Quality of Life Decreased in Adolescents With Back Pain? Spine. 2018; 43(14): E822-E829.

Kumagai G, Wada K, Tanaka T, Kudo H, Asari T, Chiba D, Ota S, Nakaji S, Ishibashi Y. Associations between neck symptoms and LDL cholesterol in a cross-sectional population-based study. J Orthop Sci. 2018; 23(2): 277-281.

Laslett LL, Menz HB, Otahal P, Pan F, Cicuttini FM, Jones G. Factors associated with prevalent and incident foot pain: data from the Tasmanian Older Adult Cohort Study. Maturitas. 2018; 118: 38-43.

Scarabottolo CC, Pinto RZ, Oliveira CB, Zanuto EF, Cardoso JR, Christofaro DGD. Back and neck pain prevalence and their association with physical inactivity domains in adolescents. Eur Spine J. 2017; 26(9): 2274-2280.

Damasceno GM, Ferreira AS, Nogueira LAC, Reis FJJ, Andrade ICS, Meziat-Filho N. Text neck and neck pain in 18-21-year-old young adults. Eur Spine J. 2018; 27(6): 1249-1254.

Ministry of Health and Welfare (Taiwan). Taiwan National Health Insurance Claims Data 2016.

Author contributions

Providing data or critical feedback on data sources

Miracle Ayomikun Adesina, Qorinah Estiningtyas Sakilah Adnani, Janardhana P Aithala, Hubert Amu, Jalal Arabloo, Ahmed Y Azzam, Maciej Banach, Amadou Barrow, Alehegn Bekele, Ajay Nagesh Bhat, Chao Cao, Akhilanand Chaurasia, Marita Cross, Garland T Culbreth, Xiaochen Dai, Huan-Ji Dong, Karsten E Dreinhoefer, Michael Ekholuenetale, Sharareh Eskandarieh, Adeniyi Francis Fagbamigbe, Jawad Fares, Ali Fatehizadeh, Alireza Feizkhah, Richard Charles Franklin, Mathewos Alemu Gebremichael, Sherief Ghozy, Mahaveer Golechha, Pouya Goleij, Lydia M Haile, Arvin Haj-Mirzaian, Netanja I. Harlianto, Simon I Hay, Amir Human Hoveidaei, Segun Emmanuel Ibitoye, Chidozie C D Iwu, Jost B. Jonas, Charity Ehimwenma Joshua, Himal Kandel, Himanshu Khajuria, Moien AB Khan, Moawiah Mohammad Khatatbeh, Sorour Khateri, Jagdish Khubchandani, Yun Jin Kim, Adnan Kisa, Jacek A Kopec, Kewal Krishan, Ambily Kuttikkattu, Savita Lasrado, Yo Han Lee, Stephen S Lim, Xuefeng Liu, Justin Lo, Lyn M March, Mohamed Kamal Mesregah, Ted R Miller, Awoke Misganaw, Shafiu Mohammed, Ali H Mokdad, Sara Momtazmanesh, Mohammad Ali Moni, Ebrahim Mostafavi, Christopher J L Murray, Ogochukwu Janet Nzoputam, Osaretin Christabel Okonji, Kanyin Liane Ong, Mayowa O Owolabi, Kevin Pacheco-Barrios, Mohammad Taha Pahlevan Fallahy, Jay Patel, Shrikant Pawar, Paolo Pedersini, Mario F P Peres, Pradhum Ram, Salman Rawaf, Umar Saeed, Monika Sawhney, Ali Shamekh, Parnian Shobeiri, Jasvinder A. Singh, Paramdeep Singh, Ambrish Singh, Anna Aleksandrovna Skryabina, Amanda E Smith, Ker-Kan Tan, Sahel Valadan Tahbaz, Tommi Juhani Vasankari, Narayanaswamy Venketasubramanian, Stein Emil Vollset, Theo Vos, Taweewat Wiangkham, Anthony D Woolf, Ai-Min Wu, Naohiro Yonemoto, and Iman Zare.

Developing methods or computational machinery

Qorinah Estiningtyas Sakilah Adnani, Hubert Amu, Aleksandr Y Aravkin, Ahmed Y Azzam, Kaleb Coberly, Garland T Culbreth, Xiaochen Dai, Ali Fatehizadeh, Sherief Ghozy, Lydia M Haile, Simon I Hay, Sorour Khateri, Adnan Kisa, Ali H Mokdad, Mohammad Ali Moni, Christopher J L Murray, Kanyin Liane Ong, Seoyeon Park, Umar Saeed, Austin E Schumacher, Jaimie D Steinmetz, Theo Vos, and Peng Zheng.

Providing critical feedback on methods or results

Mitra Abbasifard, Isaac Yeboah Addo, Miracle Ayomikun Adesina, Qorinah Estiningtyas Sakilah Adnani, Janardhana P Aithala, Yousef Alimohamadi, Sohrab Amiri, Hubert Amu, Jalal Arabloo, Mohammad Asghari-Jafarabadi, Gamechu Hunde Atomsa, Sina Azadnajafabad, Ahmed Y Azzam, Soroush Baghdadi, Saliu A Balogun, Asaminew Birhanu Balta, Maciej Banach, Morteza Banakar, Amadou Barrow, Alehegn Bekele, Isabela M Bensenor, Pankaj Bhardwaj, Ajay Nagesh Bhat, Awraris Hailu Bilchut, Andrew M Briggs,

Rachelle Buchbinder, Chao Cao, Akhilanand Chaurasia, Steffan Wittrup McPhee Christensen, Marita Cross, Garland T Culbreth, Omid Dadras, Xiaochen Dai, Katie de Luca, Azizallah Dehghan, Huan-Ji Dong, Karsten E Dreinhoefer, Michael Ekholuenetale, Muhammed Elhadi, James M Elliott, Habitu Birhan Eshetu, Sharareh Eskandarieh, Farshid Etaee, Adeniyi Francis Fagbamigbe, Jawad Fares, Ali Fatehizadeh, Alireza Feizkhah, Nuno Ferreira, Florian Fischer, Richard Charles Franklin, Balasankar Ganesan, Mathewos Alemu Gebremichael, Urge Gerema, Ali Gholami, Sherief Ghozy, Tiffany K Gill, Mahaveer Golechha, Davide Golinelli, Simon Matthew Graham, Lydia M Haile, Netanja I. Harlianto, Jan Hartvigsen, Mohammad Hasanian, Mohammed Bheser Hassen, Simon I Hay, Jeffrey J Hebert, Golnaz Heidari, Amir Human Hoveidaei, Alexander Kevin Hsiao, Segun Emmanuel Ibitoye, Chidozie C D Iwu, Louis Jacob, Yingzhao Jin, Jost B. Jonas, Charity Ehimwenma Joshua, Himal Kandel, Yousef Saleh Khader, Himanshu Khajuria, Ejaz Ahmad Khan, Moien AB Khan, Moawiah Mohammad Khatatbeh, Sorour Khateri, Mohammad Saeid Khonji, Jagdish Khubchandani, Yun Jin Kim, Adnan Kisa, Ali-Asghar Kolahi, Hamid Reza Koohestani, Deborah R Kopansky-Giles, Jacek A Kopec, Kewal Krishan, Mohammed Kuddus, Ambily Kuttikkattu, Savita Lasrado, Yo Han Lee, Samson Mideksa Legesse, Stephen S Lim, Xuefeng Liu, Justin Lo, Narges Malih, Shisir Prasad Manandhar, Lyn M March, Elezebeth Mathews, Mohamed Kamal Mesregah, Tomislav Mestrovic, Awoke Misganaw, Esmaeil Mohammadi, Shafiu Mohammed, Ali H Mokdad, Sara Momtazmanesh, Mohammad Ali Moni, Ebrahim Mostafavi, Christopher J L Murray, Tapas Sadasivan Nair, Seyed Aria Nejadghaderi, Ogochukwu Janet Nzoputam, Osaretin Christabel Okonji, Kanyin Liane Ong, Mayowa O Owolabi, Kevin Pacheco-Barrios, Mohammad Taha Pahlevan Fallahy, Seoyeon Park, Jay Patel, Shrikant Pawar, Paolo Pedersini, Mario F P Peres, Ionela-Roxana Petcu, Mohammadreza Pourahmadi, Pradhum Ram, Mohammad-Mahdi Rashidi, Salman Rawaf, Negar Rezaei, Nazila Rezaei, Umar Saeed, Fatemeh Saheb Sharif-Askari, Monika Sawhney, Mahan Shafie, Saeed Shahabi, Ataollah Shahbandi, Ali Shamekh, Saurab Sharma, Rahman Shiri, Parnian Shobeiri, Ehsan Sinaei, Jasvinder A. Singh, Paramdeep Singh, Ambrish Singh, Anna Aleksandrovna Skryabina, Amanda E Smith, Jaimie D Steinmetz, Mohammad Tabish, Ker-Kan Tan, Masresha Derese Tegegne, Samar Tharwat, Julia M Treleaven, Seyed Mohammad Vahabi, Sahel Valadan Tahbaz, Narayanaswamy Venketasubramanian, Stein Emil Vollset, Theo Vos, Yuan-Pang Wang, Taweewat Wiangkham, Anthony D Woolf, Ai-Min Wu, Naohiro Yonemoto, Moein Zangiabadian, and Dawit T Zemedikun.

Drafting the work or revising it critically for important intellectual content

Zeinab Abbasi-Kangevari, Isaac Yeboah Addo, Qorinah Estiningtyas Sakilah Adnani, Janardhana P Aithala, Fadwa Alhalaiqa Naji Alhalaiqa, Sohrab Amiri, Hubert Amu, Benny Antony, Jalal Arabloo, Mohammad Asghari-Jafarabadi, Gamechu Hunde Atomsa, Sina Azadnajafabad, Ahmed Y Azzam, Soroush Baghdadi, Saliu A Balogun, Asaminew Birhanu Balta, Maciej Banach, Morteza Banakar, Amadou Barrow, Azadeh Bashiri, Alehegn Bekele, Isabela M Bensenor, Neil Betteridge, Ajay Nagesh Bhat, Awraris Hailu Bilchut, Andrew M Briggs, Peter M Brooks, Chao Cao, Jesus Lorenzo Chirinos-Caceres, Steffan Wittrup

McPhee Christensen, Ewerton Cousin, Marita Cross, Garland T Culbreth, Katie de Luca, Karsten E Dreinhoefer, Muhammed Elhadi, James M Elliott, Sharareh Eskandarieh, Farshid Etaee, Adeniyi Francis Fagbamigbe, Jawad Fares, Ali Fatehizadeh, Nuno Ferreira, Manuela L Ferreira, Florian Fischer, Balasankar Ganesan, Urge Gerema, Ali Gholami, Sherief Ghozy, Tiffany K Gill, Davide Golinelli, Simon Matthew Graham, Lydia M Haile, Arvin Haj-Mirzaian, Netanja I. Harlianto, Jan Hartvigsen, Simon I Hay, Jeffrey J Hebert, Golnaz Heidari, Amir Human Hoveidaei, Alexander Kevin Hsiao, Segun Emmanuel Ibitoye, Chidozie C D Iwu, Louis Jacob, Manthan Dilipkumar Janodia, Jost B. Jonas, Himal Kandel, Yousef Saleh Khader, Himanshu Khajuria, Ejaz Ahmad Khan, Moien AB Khan, Moawiah Mohammad Khatatbeh, Sorour Khateri, Hamid Reza Khayat Kashani, Jagdish Khubchandani, Adnan Kisa, Jacek A Kopec, Kewal Krishan, Mohammed Kuddus, Savita Lasrado, Samson Mideksa Legesse, Justin Lo, Lyn M March, Elezebeth Mathews, Mohamed Kamal Mesregah, Tomislav Mestrovic, Ted R Miller, Seyed Peyman Mirghaderi, Awoke Misganaw, Esmaeil Mohammadi, Shafiu Mohammed, Ali H Mokdad, Sara Momtazmanesh, Mohammad Ali Moni, Ebrahim Mostafavi, Christopher J L Murray, Seyed Aria Nejadghaderi, Ogochukwu Janet Nzoputam, In-Hwan Oh, Osaretin Christabel Okonji, Mayowa O Owolabi, Kevin Pacheco-Barrios, Mohammad Taha Pahlevan Fallahy, Jay Patel, Shrikant Pawar, Paolo Pedersini, Mario F P Peres, Ionela-Roxana Petcu, Mohammadreza Pourahmadi, Ibrahim Qattea, Pradhum Ram, Salman Rawaf, Nazila Rezaei, Umar Saeed, Fatemeh Saheb Sharif-Askari, Saina Salahi, Mahan Shafie, Saeed Shahabi, Saurab Sharma, Parnian Shobeiri, Jasvinder A. Singh, Paramdeep Singh, Ambrish Singh, Anna Aleksandrovna Skryabina, Amanda E Smith, Mohammad Tabish, Ker-Kan Tan, Masresha Derese Tegegne, Samar Tharwat, Julia M Treleaven, Sahel Valadan Tahbaz, Tommi Juhani Vasankari, Narayanaswamy Venketasubramanian, Stein Emil Vollset, Theo Vos, David M Walton, Yuan-Pang Wang, Taweewat Wiangkham, Anthony D Woolf, Ai-Min Wu, Naohiro Yonemoto, and Iman Zare.

Managing the estimation or publications process

Ahmed Y Azzam, Marita Cross, James M Elliott, Ali Fatehizadeh, Hailey Hagins, Simon I Hay, Samson Mideksa Legesse, Ali H Mokdad, Christopher J L Murray, Jaimie D Steinmetz, Theo Vos, and Ai-Min Wu.
